# Supplementary material for: Is the outcome of fitting hearing aids to adults affected by whether an audiogram-based prescription formula is individually applied? A systematic review protocol
Source: BMJ Open. 2021 Aug 2;11(8):e045899. doi: 10.1136/bmjopen-2020-045899 (PMC8330563; doi:10.1136/bmjopen-2020-045899)
Supplement: Supplementary data [file bmjopen-2020-045899supp001.pdf]

Appendix 1: search protocols

PubMed:

(("Hearing Loss"[Mesh] OR "hearing loss"[tw] OR "hearing losses"[tw] OR "Hearing Disorders"[Mesh] OR "hearing disorder"[tw] OR "hearing disorders"[tw] OR "hearing problem"[tw] OR "hearing problems"[tw] OR "hearing impaired"[tw] OR "hearing difficulties"[tw] OR "hearing difficulty"[tw] OR "ANSD"[tw] OR "auditory neuropathy"[tw]) AND ("Hearing Aids"[Mesh] OR "hearing aid"[tw] OR "hearing aids"[tw] OR "hearing device"[tw] OR "hearing devices"[tw] OR "acoustic aid"[tw] OR "acoustic aids"[tw] OR " hearing instrument"[tw] OR " hearing instruments"[tw] OR "over-the-counter hearing"[tw] OR "over the counter hearing "[tw] OR "direct-to-consumer hearing"[tw] OR "direct to consumer hearing"[tw] OR "self-fitting hearing"[tw] OR "self fitting hearing "[tw] OR "personal sound amplification"[tw]) AND ("real ear measurement"[tw] OR "real ear measurements"[tw] OR "real ear measure"[tw] OR "real ear measures"[tw] OR "real-ear measurement"[tw] OR "real-ear measurements"[tw] OR "real-ear measure"[tw] OR "real-ear measures"[tw] OR "REM"[tw] OR "REMs"[tw] OR "PMM"[tw] OR "PMMs"[tw] OR "probe microphone"[tw] OR "probe microphones"[tw] OR "probe mic"[tw] OR "probe mics"[tw] OR "probe microphone measurement"[tw] OR "probe microphone measurements"[tw] OR "probe microphone measure"[tw] OR "probe microphone measures"[tw] OR "probemicrophone"[tw] OR "probe-microphones"[tw] OR "probe-microphone measurement"[tw] OR "probe-microphone measurements"[tw] OR "probe-microphone measure"[tw] OR "probe-microphone measures"[tw] OR "real-ear verification"[tw] OR "audiology best practices"[tw] OR "audiology best practice"[tw] OR "best practices"[tw] OR "best practice"[tw] OR "clinician-driven"[tw] OR "physician-driven"[tw] OR "doctor-driven"[tw] OR "prescriptive methods"[tw] OR "prescription methods"[tw] OR "prescriptive method*"[tw] OR "prescription method*"[tw] OR "formula"[tw] OR "formulae"[tw]))

MEDLINE (OVID)

((exp "Hearing Loss"/ OR "hearing loss".mp OR "hearing losses".mp OR exp "Hearing Disorders"/ OR "hearing disorder".mp OR "hearing disorders".mp OR "hearing problem".mp OR "hearing problems".mp OR "hearing impaired".mp OR "hearing difficulties".mp OR "hearing difficulty".mp OR "ANSD".mp OR "auditory neuropathy".mp) AND (exp "Hearing Aids"/ OR "hearing aid".mp OR "hearing aids".mp OR "hearing device".mp OR "hearing devices".mp OR "acoustic aid".mp OR "acoustic aids".mp OR " hearing instrument".mp OR " hearing instruments".mp OR "over-the-counter hearing".mp OR "over the counter hearing ".mp OR "direct-to-consumer hearing".mp OR "direct to consumer hearing".mp OR "self-fitting hearing".mp OR "self fitting hearing ".mp OR "personal sound amplification".mp) AND ("real ear measurement".mp OR "real ear measurements".mp OR "real ear measure".mp OR "real ear measures".mp OR "real-ear measurement".mp OR "real-ear measurements".mp OR "real-ear measure".mp OR "real-ear measures".mp OR "REM".mp OR "REMs".mp OR "PMM".mp OR "PMMs".mp OR "probe microphone".mp OR "probe microphones".mp OR "probe mic".mp OR "probe mics".mp OR "probe microphone measurement".mp OR "probe microphone measurements".mp OR "probe microphone measure".mp OR "probe microphone measures".mp OR "probemicrophone".mp OR "probe-microphones".mp OR "probe-microphone measurement".mp OR "probe-microphone measurements".mp OR "probe-microphone measure".mp OR "probe-microphone measures".mp OR "real-ear verification".mp OR "audiology best practices".mp OR "audiology best practice".mp OR "best practices".mp OR "best practice".mp OR "clinician-driven".mp OR "physician-driven".mp OR "doctor-driven".mp OR "prescriptive methods".mp OR "prescription methods".mp OR "prescriptive method*".mp OR "prescription method*".mp OR "formula".mp OR "formulae".mp))

Embase (OVID)

((exp "Hearing Impairment"/ OR "hearing loss".mp OR "hearing losses".mp OR exp "Hearing Disorder"/ OR "hearing disorder".mp OR "hearing disorders".mp OR "hearing problem".mp OR "hearing problems".mp OR "hearing impaired".mp OR "hearing difficulties".mp OR "hearing difficulty".mp OR "ANSD".mp OR "auditory neuropathy".mp) AND (exp "Hearing Aid"/ OR "hearing aid".mp OR "hearing aids".mp OR "hearing device".mp OR "hearing devices".mp OR "acoustic aid".mp OR "acoustic aids".mp OR " hearing instrument".mp OR " hearing instruments".mp OR "over-the-counter hearing".mp OR "over the counter hearing ".mp OR "direct-to-consumer hearing".mp OR "direct to consumer hearing".mp OR "self-fitting hearing".mp OR "self fitting hearing ".mp OR "personal sound amplification".mp) AND ("real ear measurement".mp OR "real ear measurements".mp OR "real ear measure".mp OR "real ear measures".mp OR "real-ear measurement".mp OR "real-ear measurements".mp OR "real-ear measure".mp OR "real-ear measures".mp OR "REM".mp OR "REMs".mp OR "PMM".mp OR "PMMs".mp OR "probe microphone".mp OR "probe microphones".mp OR "probe mic".mp OR "probe mics".mp OR "probe microphone measurement".mp OR "probe microphone measurements".mp OR "probe microphone measure".mp OR "probe microphone measures".mp OR "probemicrophone".mp OR "probe-microphones".mp OR "probe-microphone measurement".mp OR "probe-microphone measurements".mp OR "probe-microphone measure".mp OR "probe-microphone measures".mp OR "real-ear verification".mp OR "audiology best practices".mp OR "audiology best practice".mp OR "best practices".mp OR "best practice".mp OR "clinician-driven".mp OR "physician-driven".mp OR "doctor-driven".mp OR "prescriptive methods".mp OR "prescription methods".mp OR "prescriptive method*".mp OR "prescription method*".mp OR "formula".mp OR "formulae".mp))

PsycINFO

TX(("Hearing Impairment" OR "hearing loss" OR "hearing losses" OR "Hearing Disorder" OR "hearing disorder" OR "hearing disorders" OR "hearing problem" OR "hearing problems" OR "hearing impaired" OR "hearing difficulties" OR "hearing difficulty" OR "ANSD" OR "auditory neuropathy") AND ("Hearing Aid" OR "hearing aid" OR "hearing aids" OR "hearing device" OR "hearing devices" OR "acoustic aid" OR "acoustic aids" OR " hearing instrument" OR " hearing instruments" OR "over-the-counter hearing" OR "over the counter hearing " OR "direct-to-consumer hearing" OR "direct to consumer hearing" OR "self-fitting hearing" OR "self fitting hearing " OR "personal sound amplification") AND ("real ear measurement" OR "real ear measurements" OR "real ear measure" OR "real ear measures" OR "real-ear measurement" OR "real-ear measurements" OR "real-ear measure" OR "real-ear measures" OR "REM" OR "REMs" OR "PMM" OR "PMMs" OR "probe microphone" OR "probe microphones" OR "probe mic" OR "probe mics" OR "probe microphone measurement" OR "probe microphone measurements" OR "probe microphone measure" OR "probe microphone measures" OR "probemicrophone" OR "probe-microphones" OR "probe-microphone measurement" OR "probe-microphone measurements" OR "probe-microphone measure" OR "probe-microphone measures" OR "real-ear verification" OR "audiology best practices" OR "audiology best practice" OR "best practices" OR "best practice" OR "clinician-driven" OR "physician-driven" OR "doctor-driven" OR "prescriptive methods" OR "prescription methods" OR "prescriptive method*" OR "prescription method*" OR "formula" OR "formulae"))

Web of Science (all databases)

TS=(("Hearing Impairment" OR "hearing loss" OR "hearing losses" OR "Hearing Disorder" OR "hearing disorder" OR "hearing disorders" OR "hearing problem" OR "hearing problems" OR "hearing impaired" OR "hearing difficulties" OR "hearing difficulty" OR "ANSD" OR "auditory neuropathy") AND ("Hearing Aid" OR "hearing aid" OR "hearing aids" OR "hearing device" OR "hearing devices" OR "acoustic aid" OR "acoustic aids" OR " hearing instrument" OR " hearing instruments" OR "over-the-counter hearing" OR "over the counter hearing " OR "direct-to-consumer hearing" OR "direct to consumer hearing" OR "self-fitting hearing" OR "self fitting hearing " OR "personal sound amplification") AND ("real ear measurement" OR "real ear measurements" OR "real ear measure" OR "real ear measures" OR "real-ear measurement" OR "real-ear measurements" OR "real-ear measure" OR "real-ear measures" OR "REM" OR "REMs" OR "PMM" OR "PMMs" OR "probe microphone" OR "probe microphones" OR "probe mic" OR "probe mics" OR "probe microphone measurement" OR "probe microphone measurements" OR "probe microphone measure" OR "probe microphone measures" OR "probemicrophone" OR "probe-microphones" OR "probe-microphone measurement" OR "probe-microphone measurements" OR "probe-microphone measure" OR "probe-microphone measures" OR "real-ear verification" OR "audiology best practices" OR "audiology best practice" OR "best practices" OR "best practice" OR "clinician-driven" OR "physician-driven" OR "doctor-driven" OR "prescriptive methods" OR "prescription methods" OR "prescriptive method*" OR "prescription method*" OR "formula" OR "formulae"))

Cochrane Library

(("Hearing Impairment" OR "hearing loss" OR "hearing losses" OR "Hearing Disorder" OR "hearing disorder" OR "hearing disorders" OR "hearing problem" OR "hearing problems" OR "hearing impaired" OR "hearing difficulties" OR "hearing difficulty" OR "ANSD" OR "auditory neuropathy") AND ("Hearing Aid" OR "hearing aid" OR "hearing aids" OR "hearing device" OR "hearing devices" OR "acoustic aid" OR "acoustic aids" OR " hearing instrument" OR " hearing instruments" OR "over-the-counter hearing" OR "over the counter hearing " OR "direct-to-consumer hearing" OR "direct to consumer hearing" OR "self-fitting hearing" OR "self fitting hearing " OR "personal sound amplification") AND ("real ear measurement" OR "real ear measurements" OR "real ear measure" OR "real ear measures" OR "real-ear measurement" OR "real-ear measurements" OR "real-ear measure" OR "real-ear measures" OR "REM" OR "REMs" OR "PMM" OR "PMMs" OR "probe microphone" OR "probe microphones" OR "probe mic" OR "probe mics" OR "probe microphone measurement" OR "probe microphone measurements" OR "probe microphone measure" OR "probe microphone measures" OR "probemicrophone" OR "probe-microphones" OR "probe-microphone measurement" OR "probe-microphone measurements" OR "probe-microphone measure" OR "probe-microphone measures" OR "real-ear verification" OR "audiology best practices" OR "audiology best practice" OR "best practices" OR "best practice" OR "clinician-driven" OR "physician-driven" OR "doctor-driven" OR "prescriptive methods" OR "prescription methods" OR "prescriptive method*" OR "prescription method*" OR "formula" OR "formulae")):ti,ab,kw

Emcare

((exp "Hearing Impairment"/ OR "hearing loss".mp OR "hearing losses".mp OR exp "Hearing Disorder"/ OR "hearing disorder".mp OR "hearing disorders".mp OR "hearing problem".mp OR "hearing problems".mp OR "hearing impaired".mp OR "hearing difficulties".mp OR "hearing difficulty".mp OR "ANSD".mp OR "auditory neuropathy".mp) AND (exp "Hearing Aid"/ OR "hearing aid".mp OR "hearing aids".mp OR "hearing device".mp OR "hearing devices".mp OR

"acoustic aid".mp OR "acoustic aids".mp OR " hearing instrument".mp OR " hearing instruments".mp OR "over-the-counter hearing".mp OR "over the counter hearing ".mp OR "direct-to-consumer hearing".mp OR "direct to consumer hearing".mp OR "self-fitting hearing".mp OR "self fitting hearing ".mp OR "personal sound amplification".mp) AND ("real ear measurement".mp OR "real ear measurements".mp OR "real ear measure".mp OR "real ear measures".mp OR "real-ear measurement".mp OR "real-ear measurements".mp OR "real-ear measure".mp OR "real-ear measures".mp OR "REM".mp OR "REMs".mp OR "PMM".mp OR "PMMs".mp OR "probe microphone".mp OR "probe microphones".mp OR "probe mic".mp OR "probe mics".mp OR "probe microphone measurement".mp OR "probe microphone measurements".mp OR "probe microphone measure".mp OR "probe microphone measures".mp OR "probemicrophone".mp OR "probe-microphones".mp OR "probe-microphone measurement".mp OR "probe-microphone measurements".mp OR "probe-microphone measure".mp OR "probe-microphone measures".mp OR "real-ear verification".mp OR "audiology best practices".mp OR "audiology best practice".mp OR "best practices".mp OR "best practice".mp OR "clinician-driven".mp OR "physician-driven".mp OR "doctor-driven".mp OR "prescriptive methods".mp OR "prescription methods".mp OR "prescriptive method*".mp OR "prescription method*".mp OR "formula".mp OR "formulae".mp))

Academic Search Premier

TI(("Hearing Impairment" OR "hearing loss" OR "hearing losses" OR "Hearing Disorder" OR "hearing disorder" OR "hearing disorders" OR "hearing problem" OR "hearing problems" OR "hearing impaired" OR "hearing difficulties" OR "hearing difficulty" OR "ANSD" OR "auditory neuropathy") AND ("Hearing Aid" OR "hearing aid" OR "hearing aids" OR "hearing device" OR "hearing devices" OR "acoustic aid" OR "acoustic aids" OR " hearing instrument" OR " hearing instruments" OR "over-the-counter hearing" OR "over the counter hearing " OR "direct-to-consumer hearing" OR "direct to consumer hearing" OR "self-fitting hearing" OR "self fitting hearing " OR "personal sound amplification") AND ("real ear measurement" OR "real ear measurements" OR "real ear measure" OR "real ear measures" OR "real-ear measurement" OR "real-ear measurements" OR "real-ear measure" OR "real-ear measures" OR "REM" OR "REMs" OR "PMM" OR "PMMs" OR "probe microphone" OR "probe microphones" OR "probe mic" OR "probe mics" OR "probe microphone measurement" OR "probe microphone measurements" OR "probe microphone measure" OR "probe microphone measures" OR "probemicrophone" OR "probe-microphones" OR "probe-microphone measurement" OR "probe-microphone measurements" OR "probe-microphone measure" OR "probe-microphone measures" OR "real-ear verification" OR "audiology best practices" OR "audiology best practice" OR "best practices" OR "best practice" OR "clinician-driven" OR "physician-driven" OR "doctor-driven" OR "prescriptive methods" OR "prescription methods" OR "prescriptive method*" OR "prescription method*" OR "formula" OR "formulae")) OR SU(("Hearing Impairment" OR "hearing loss" OR "hearing losses" OR "Hearing Disorder" OR "hearing disorder" OR "hearing disorders" OR "hearing problem" OR "hearing problems" OR "hearing impaired" OR "hearing difficulties" OR "hearing difficulty" OR "ANSD" OR "auditory neuropathy") AND ("Hearing Aid" OR "hearing aid" OR "hearing aids" OR "hearing device" OR "hearing devices" OR "acoustic aid" OR "acoustic aids" OR " hearing instrument" OR " hearing instruments" OR "over-the-counter hearing" OR "over the counter hearing " OR "direct-to-consumer hearing" OR "direct to consumer hearing" OR "self-fitting hearing" OR "self fitting hearing " OR "personal sound amplification") AND ("real ear measurement" OR "real ear measurements" OR "real ear measure" OR "real ear measures" OR "real-ear measurement" OR "real-ear measurements" OR "real-ear measure" OR "real-ear measures" OR "REM" OR "REMs" OR "PMM" OR "PMMs" OR "probe microphone" OR "probe microphones" OR "probe mic" OR "probe mics" OR "probe microphone measurement" OR "probe microphone measurements" OR "probe microphone measure" OR "probe microphone

measures" OR "probemicrophone" OR "probe-microphones" OR "probe-microphone measurement" OR "probe-microphone measurements" OR "probe-microphone measure" OR "probe-microphone measures" OR "real-ear verification" OR "audiology best practices" OR "audiology best practice" OR "best practices" OR "best practice" OR "clinician-driven" OR "physician-driven" OR "doctor-driven" OR "prescriptive methods" OR "prescription methods" OR "prescriptive method*" OR "prescription method*" OR "formula" OR "formulae")) OR KW(("Hearing Impairment" OR "hearing loss" OR "hearing losses" OR "Hearing Disorder" OR "hearing disorder" OR "hearing disorders" OR "hearing problem" OR "hearing problems" OR "hearing impaired" OR "hearing difficulties" OR "hearing difficulty" OR "ANSD" OR "auditory neuropathy") AND ("Hearing Aid" OR "hearing aid" OR "hearing aids" OR "hearing device" OR "hearing devices" OR "acoustic aid" OR "acoustic aids" OR " hearing instrument" OR " hearing instruments" OR "over-the-counter hearing" OR "over the counter hearing " OR "direct-to-consumer hearing" OR "direct to consumer hearing" OR "self-fitting hearing" OR "self fitting hearing " OR "personal sound amplification") AND ("real ear measurement" OR "real ear measurements" OR "real ear measure" OR "real ear measures" OR "real-ear measurement" OR "real-ear measurements" OR "real-ear measure" OR "real-ear measures" OR "REM" OR "REMs" OR "PMM" OR "PMMs" OR "probe microphone" OR "probe microphones" OR "probe mic" OR "probe mics" OR "probe microphone measurement" OR "probe microphone measurements" OR "probe microphone measure" OR "probe microphone measures" OR "probemicrophone" OR "probe-microphones" OR "probe-microphone measurement" OR "probe-microphone measurements" OR "probe-microphone measure" OR "probe-microphone measures" OR "real-ear verification" OR "audiology best practices" OR "audiology best practice" OR "best practices" OR "best practice" OR "clinician-driven" OR "physician-driven" OR "doctor-driven" OR "prescriptive methods" OR "prescription methods" OR "prescriptive method*" OR "prescription method*" OR "formula" OR "formulae")) OR AB(("Hearing Impairment" OR "hearing loss" OR "hearing losses" OR "Hearing Disorder" OR "hearing disorder" OR "hearing disorders" OR "hearing problem" OR "hearing problems" OR "hearing impaired" OR "hearing difficulties" OR "hearing difficulty" OR "ANSD" OR "auditory neuropathy") AND ("Hearing Aid" OR "hearing aid" OR "hearing aids" OR "hearing device" OR "hearing devices" OR "acoustic aid" OR "acoustic aids" OR " hearing instrument" OR " hearing instruments" OR "over-the-counter hearing" OR "over the counter hearing " OR "direct-to-consumer hearing" OR "direct to consumer hearing" OR "self-fitting hearing" OR "self fitting hearing " OR "personal sound amplification") AND ("real ear measurement" OR "real ear measurements" OR "real ear measure" OR "real ear measures" OR "real-ear measurement" OR "real-ear measurements" OR "real-ear measure" OR "real-ear measures" OR "REM" OR "REMs" OR "PMM" OR "PMMs" OR "probe microphone" OR "probe microphones" OR "probe mic" OR "probe mics" OR "probe microphone measurement" OR "probe microphone measurements" OR "probe microphone measure" OR "probe microphone measures" OR "probemicrophone" OR "probe-microphones" OR "probe-microphone measurement" OR "probe-microphone measurements" OR "probe-microphone measure" OR "probe-microphone measures" OR "real-ear verification" OR "audiology best practices" OR "audiology best practice" OR "best practices" OR "best practice" OR "clinician-driven" OR "physician-driven" OR "doctor-driven" OR "prescriptive methods" OR "prescription methods" OR "prescriptive method*" OR "prescription method*" OR "formula" OR "formulae"))
