# Supplementary material for: Is the outcome of fitting hearing aids to adults affected by whether an audiogram-based prescription formula is individually applied? A systematic review protocol
Source: BMJ Open. 2021 Aug 2;11(8):e045899. doi: 10.1136/bmjopen-2020-045899 (PMC8330563; doi:10.1136/bmjopen-2020-045899)
Supplement: Supplementary data [file bmjopen-2020-045899supp002.pdf]

Appendix 2: Data items

General information	Method	Participants	Intervention	Outcome	Funding source	Declaration of interest	Additional comments
Title<br>Authors<br>Publication year	Study design<br>Conditions and the full study durations<br>Sequence generation<br>Sequence concealment<br>Blinding<br>Power analysis	Sample size<br>Country / Setting<br>Age<br>Sex<br>Inclusion and exclusion criteria	Intervention group<br>Comparator group	Primary outcome<br>Secondary outcome(s)<br>Adverse events			
