# Supplementary material for: Is the outcome of fitting hearing aids to adults affected by whether an audiogram-based prescription formula is individually applied? A systematic review protocol
Source: BMJ Open. 2021 Aug 2;11(8):e045899. doi: 10.1136/bmjopen-2020-045899 (PMC8330563; doi:10.1136/bmjopen-2020-045899)
Supplement: Author's manuscript [file bmjopen-2020-045899.draft_revisions.pdf]

BMJ Open is committed to open peer review. As part of this commitment we make the peer review history of every article we publish publicly available.

When an article is published we post the peer reviewers' comments and the authors' responses online. We also post the versions of the paper that were used during peer review. These are the versions that the peer review comments apply to.

The versions of the paper that follow are the versions that were submitted during the peer review process. They are not the versions of record or the final published versions. They should not be cited or distributed as the published version of this manuscript.

BMJ Open is an open access journal and the full, final, typeset and author-corrected version of record of the manuscript is available on our site with no access controls, subscription charges or pay-per-view fees (http://bmjopen.bmj.com).

If you have any questions on BMJ Open's open peer review process please email info.bmjopen@bmj.com

BMJ Open

Is the outcome better for adults when hearing aids are programmed using audiogram-based prescription formulae? A systematic review protocol.

Journal:	*BMJ Open*
Manuscript ID	bmjopen-2020-045899
Article Type:	Protocol
Date Submitted by the Author:	17-Oct-2020
Complete List of Authors:	Almufarrij, Ibrahim; The University of Manchester, Manchester Centre for Audiology and Deafness, School of Health Sciences; King Saud University, Department of Rehabilitation Sciences<br>Dillon, Harvey; The University of Manchester, Manchester Centre for Audiology and Deafness, School of Health Sciences; Macquarie University, Department of Linguistics<br>Munro, Kevin; The University of Manchester, Manchester Centre for Audiology and Deafness, School of Health Sciences; Manchester University NHS Foundation Trust, Manchester University Hospitals NHS Foundation Trust, Manchester Academic Health Science Centre,
Keywords:	Audiology < OTOLARYNGOLOGY, Neurotology < OTOLARYNGOLOGY, Adult otolaryngology < OTOLARYNGOLOGY

SCHOLARONE™
Manuscripts

I, the Submitting Author has the right to grant and does grant on behalf of all authors of the Work (as defined in the below author licence), an exclusive licence and/or a non-exclusive licence for contributions from authors who are: i) UK Crown employees; ii) where BMJ has agreed a CC-BY licence shall apply, and/or iii) in accordance with the terms applicable for US Federal Government officers or employees acting as part of their official duties; on a worldwide, perpetual, irrevocable, royalty-free basis to BMJ Publishing Group Ltd ("BMJ") its licensees and where the relevant Journal is co-owned by BMJ to the co-owners of the Journal, to publish the Work in this journal and any other BMJ products and to exploit all rights, as set out in our licence.

The Submitting Author accepts and understands that any supply made under these terms is made by BMJ to the Submitting Author unless you are acting as an employee on behalf of your employer or a postgraduate student of an affiliated institution which is paying any applicable article publishing charge ("APC") for Open Access articles. Where the Submitting Author wishes to make the Work available on an Open Access basis (and intends to pay the relevant APC), the terms of reuse of such Open Access shall be governed by a Creative Commons licence – details of these licences and which Creative Commons licence will apply to this Work are set out in our licence referred to above.

Other than as permitted in any relevant BMJ Author's Self Archiving Policies, I confirm this Work has not been accepted for publication elsewhere, is not being considered for publication elsewhere and does not duplicate material already published. I confirm all authors consent to publication of this Work and authorise the granting of this licence.

For peer review only

Is the outcome better for adults when hearing aids are programmed using audiogram-based prescription formulae? A systematic review protocol.

Authors: Ibrahim Almufarrij[1, 2], Harvey Dillon[1,4] and Kevin J Munro[1, 3]

Author affiliations

[1] Manchester Centre for Audiology and Deafness, School of Health Sciences, University of Manchester and the Manchester Academic Health Science Centre, UK

[2] Department of Rehabilitation Sciences, College of Applied Medical Sciences, King Saud University, Riyadh, Kingdom of Saudi Arabia

[3] Manchester University Hospitals NHS Foundation Trust, Manchester Academic Health Science Centre, UK

[4] Department of Linguistics, Macquarie University, Sydney, Australia

Corresponding author: Ibrahim Almufarrij, Manchester Centre for Audiology and Deafness, University of Manchester, Manchester, M13 9PL, United Kingdom. E-mail: Email: ialmufarrij@ksu.edu.sa

For peer review only

ABSTRACT

Introduction: Hearing aids are typically programmed using the individual's audiometric thresholds. Developments in technology have resulted in a new category of direct-to-consumer devices, which are not programmed using the individual's audiometric thresholds. This review aims to identify whether programming hearing aids using the individual's audiogram-based prescription results in better outcomes for adults with hearing loss.

Methods and analysis: The methods of this review are reported in line with Preferred Reporting Items for Systematic Reviews and Meta-Analyses Protocols guidelines. More than eight different databases will be systematically searched to identify published, completed but yet-to-be published and ongoing studies. The population of interest of this review will be adults with any degree or type of hearing loss. The studies should compare hearing aids programmed using an audiogram-based prescription (and verified in the real ear) to those not programmed on the basis of the individual's audiogram. The primary outcome of interest is consumers' listening preferences. Hearing-specific health-related quality of life, self-reported listening ability, speech intelligibility of words and sentences in quiet and noisy situations, sound quality ratings and adverse events are the secondary outcomes of interest. Both randomised and non-randomised controlled trials will be included. The quality of each individual study and the overall evidence will be assessed using Down and Black's checklist and the Grading of Recommendations, Assessment, Development and Evaluations tool, respectively.

Ethics and dissemination: We will only retrieve and analyse data from published studies, so no ethical approval is required. The review findings will be published in a peer-reviewed journal and presented at scientific conferences.

PROSPERO registration number: CRD42020197232

For peer review only - http://bmjopen.bmj.com/site/about/guidelines.xhtml

Strengths and limitations of the study:

- This review, the first on this topic, compares the outcomes of programming hearing aids using audiogram-based prescriptions with those not programmed on the basis of the individual's audiogram.
- The findings of this review will have implications for hearing aid provision pathways and the emerging category of direct-to-consumer devices, which consumers can self-fit without using their audiometric thresholds.
- Studies that compared programming approaches amongst children will be excluded, as children may not be capable of fine-tuning their own hearing devices.
- We will include only those studies that compare programming approaches using the same hearing device, as differences in technology, features and appearance could serve as serious confounding variables.
- Studies published in non-peer-reviewed journals will be excluded.

INTRODUCTION

Hearing loss is a debilitating health condition that affects more than 450 million individuals worldwide [1]. It is also the most common cause for years lived with disability [2]. Hearing loss can directly affect people's ability to communicate, which can consequently lead to depression and poor social interaction [3]. It is also associated with a reduced quality of life and an increased risk of dementia [4]. Hearing loss can be partially and successfully mitigated through the use of complex electroacoustic amplification devices known as hearing aids [5].

Hearing aids are the primary and the most common clinical intervention for hearing loss because they are very effective in improving hearing-related quality of life [6, 7]. The ultimate goals for hearing aids are to restore the audibility of soft level sounds, maximise the intelligibility of conversational-level sounds and ensure that loud sounds are within comfortable levels [8].

In the early 1940s and 1950s, the most controversial aspects of clinical audiology were hearing aid selection and fitting, as experts could not agree upon the most appropriate approaches [9]. Since this period, prescriptive approaches have evolved to provide recommended gains for each audiometric frequency and input level. The concept of hearing aid prescriptions goes back to the early 1940s when Jones and Knudsen (1940) developed the audiogram mirroring prescription procedure, in which every decibel (dB) of loss was compensated with an additional dB of gain [10]. The prescription was then developed by Watson and Knudsen (1940) to incorporate the individual's most comfortable level [11]. Shortly after, Lybarger (1944) developed the half-gain rule, in which every additional 2 dB of

For peer review only - http://bmjopen.bmj.com/site/about/guidelines.xhtml

hearing loss were compensated with an additional 1 dB of gain [12]. Since then, many other prescription procedures have been developed, but very few of them have been comprehensively studied and validated. The invaluable contribution of Denis Byrne (who is known as the father of prescriptive hearing aid fitting) to the development of prescription procedures has led to the indubitable universal acceptance of hearing aid prescriptions [13].

At present, several acoustic laboratories worldwide have developed validated audiogram-based prescription formulae, such as National Acoustic Laboratories Non-Linear (NAL-NL1 and NAL-NL2) [14, 15] and Desired Sensation Level (DSL5.0) [16]. These prescription formulae aimed to provide the optimal amount of amplification for each audiometric frequency [17]. Such prescriptions were revised and updated multiple times based on a combination of theoretical derivation and empirical data [18].

Rationale

Numerous direct-to-consumer hearing devices have been mass-produced and marketed to customers. Although these hearing devices vary considerably in quality, a few have comparable electroacoustics to conventional hearing aids that are programmed and fitted by audiologists [19]. Moreover, some of these hearing devices allow consumers to use their smartphone or remote controls to adjust the amplification characteristics according to their preferences [20]. Allowing users to take control of their amplification characteristics may help them to achieve a response that is better for them than one based on prescription targets that reflect what is optimal for an average person who has the same audiogram as that individual user. Alternatively, imprecision in adjustments made by the individual, or an inadequately adjustable hearing aid, may result in poorer outcomes than for hearing aids adjusted by a clinician on the basis of the individual's audiogram. In addition, individuals with hearing loss may prefer different amplification characteristics for different acoustic environments [21, 22], in ways that vary beyond the variation prescribed by existing non-linear prescription formula.

Objectives

It is unknown whether the use of individually prescribed amplification characteristics, using audiogram-based prescription formulae, and verified using real-ear probe microphone measurements, provides better outcomes than using amplification characteristics that are not prescribed on basis of the individual's audiogram (e.g., users adjust the amplification characteristics of their device based on their listening needs). Mueller (2005) systematically reviewed studies that compared audiogram-based prescriptions with each other or with the same prescription but with the user's adjustment to the overall gain [23]. Direct-to-consumer hearing devices that allow users to fine-tune the amplification characteristics without audiometric thresholds were not widely available at the time of Mueller's

For peer review only - http://bmjopen.bmj.com/site/about/guidelines.xhtml

review. Thus, this review aims to identify whether the outcomes for adults are better when hearing aids are programmed using an audiogram-based prescription formulae.

METHOD AND ANALYSIS

This review protocol was pre-registered in International Prospective Register of Systematic Reviews. The method of this systematic review are reported in line with the Preferred Reporting Items for Systematic Review and Meta-Analyses (PRISMA-P) guidelines [24].

Eligibility criteria

The eligibility criteria of this review are specified and described in line with the participants, interventions, comparators, outcomes and study designs (PICOS) criteria.

Participants

Adults (≥ 18 years) with any defined degree and type of hearing loss will be included. If only qualitative descriptions of age and hearing thresholds are reported in a trial, then the study will still be included. Studies that include both adults and children will be included if the results were separately analysed and reported. In addition, studies that include participants with normal hearing and hearing loss will not be included unless they were analysed independently.

Interventions

The intervention of interest is any amplification device (i.e. conventional hearing aids or direct-to-consumer hearing devices) that has not been programmed (using either a verified or a manufacturer proprietary prescription formula) using the individual user's hearing thresholds. Amplification devices pre-programmed for generic mild or moderate hearing loss will be included, unless the experimental protocol tested the device only on people selected because they have audiograms that closely matched the generic hearing loss target(s).

Comparators

Comparators will include any amplification device (i.e. conventional hearing aids or direct-to-consumer hearing devices) programmed for an audiogram-based prescription target (e.g. NAL-NL2) and verified using a real-ear measurement system. Simulated hearing aids, implantable devices, assistive listening devices and bone conduction hearing devices will be excluded.

For peer review only - http://bmjopen.bmj.com/site/about/guidelines.xhtml

Outcomes

The primary outcome of this review is the participant's listening preference. Secondary outcomes will include hearing-specific health-related quality of life (e.g. Hearing Handicap Inventory for the Elderly [25]), self-reported listening ability (e.g. The Speech, Spatial and Qualities of Hearing Scale [26]), speech intelligibility of words and sentences in quiet and noisy situations, sound quality ratings and adverse events (e.g. discomfort, hearing aid rejection and noise-induced hearing loss). Studies that only quantify the deviation from the prescriptive targets will be excluded. Studies that used only predicted speech intelligibility (speech intelligibility index) as an outcome will be also be excluded.

Study designs

We will include both randomised and non-randomised controlled trials, and cross-over designs. Conference abstracts, book chapters and theses will be excluded. Case reports, reviews and clinical guidelines will also be excluded.

Information sources

A systematic search strategy will be conducted to identify published, concluded but unpublished and ongoing experiments. The following databases will be searched: EMBASE, MEDLINE, PubMed, PsycINFO, Web of Science, Cochrane Library, Emcare and Academic Search Premier. All databases will be searched on the same day with no search restrictions in relation to the publication's year, status and language.

The reference lists of the included studies will be scanned to identify other relevant studies. We will also track the citation of the included studies using Google Scholar to identify additional relevant articles.

Search strategy

A medical information specialist will develop the search protocol. The search terms were developed based on free text, expert opinions and controlled terms (e.g. Medical Subject Headings). The search protocol for each of the included databases is reported in Appendix 1.

Study records

Data management

The search results records will be extracted to EndNote X9 Reference Management software (Clarivate Analytics, 2018) to remove duplicated records. Study details (i.e. authors, publication year,

For peer review only - http://bmjopen.bmj.com/site/about/guidelines.xhtml

titles and abstracts) will then be transformed into a Microsoft Excel (2016) spreadsheet so that they can be easily assessed for inclusion and exclusion criteria.

Selection process

Two of the review team will independently assess the title and abstract of all retrieved studies to determine their eligibility for inclusion. The reason for excluding any article will be documented. Disagreements will be resolved by discussion or by consulting the third author. Articles that meet the inclusion criteria will be retrieved for a full inspection. The first and second authors will then screen the retrieved records against the eligibility criteria and disagreements will be resolved by discussion with the third author. The study selection process along with the main reasons for exclusion will be illustrated in a PRISMA flow diagram.

Data collection process and data items

The first author will extract the data from all eligible studies. Another member of the review team will independently extract a small proportion of data to check for consistency. Any disagreements will be resolved by arbitration or by consulting the third author. An adapted version of the Cochrane Handbook data extraction form will be used to extract the data from the included studies. The main items of the data extraction are summarised in Appendix 2. When necessary, an extraction tool (e.g. WebPlotDigitizer) will be used to extract data that are only available in figures and graphs.

Risk of bias in individual studies

The methodological quality of the studies will be assessed using the Downs and Black (1998) checklist because it is easy to use and has acceptable validity and reliability. This tool provides numeric scores for each of the following domains: study quality, external validity, study bias, confounding and selection bias as well as power of the study. In this review, the score for the power domain (i.e. question number 27) will be reduced from three points to one point because there is a dearth of knowledge about the clinically important differences in hearing aid outcomes. The full score for this domain will be awarded if sample size calculation was made and a score of zero will be awarded if it was not. However, if no power analysis was performed but the sample size is commensurate with the other studies for which one was performed, the study will not be penalised. Thus, the maximum possible score will be 28 and the quality of each study will be categorised as excellent (26–28), good (20–25), fair (15–19) or poor quality (<14). The assessment of the risk of bias will be carried out independently by all authors. Disagreements will be resolved by discussion or decision by majority.

For peer review only - http://bmjopen.bmj.com/site/about/guidelines.xhtml

Data synthesis

The data will be synthesised into a meta-analysis where possible. For between-group studies, the mean difference (when the same continuous outcomes are used across studies) or the standardised mean difference (when different continuous outcomes are used) will be calculated along with their 95% confidence interval (CI). The risk ratio and its 95% CI will be calculated for dichotomous outcomes. For crossover design studies, the effect size and its 95% CI will be calculated in accordance with Cochrane handbook recommendations, which usually require the calculation of the correlation coefficient between the evaluated interventions. A fixed-effect meta-analysis will be computed whenever the statistical heterogeneity is low; otherwise, the data will be synthesised using a random-effect meta-analysis. The generic inverse of variance approach will be used to weight each study and hence compute the effect estimate and its 95% CI. The data for each meta-analysis will be presented in a forest plot.

Asymmetrical distribution of outcomes will be assessed where either the lowest or the highest possible values of a used scale are available. The assessment involves subtracting the lowest possible value from the mean (or vice versa with the highest possible value) and dividing this by the standard deviation (SD). When the observed ratio is less than 1, there is strong evidence of a skewed distribution. A ratio between 1 and 2 also suggests a skewed distribution. Where possible, appropriate transformation methods will be used to transform and normalise the distribution of skewed data.

The data will be quantitatively instead of qualitatively synthesised when a meta-analysis is considered to be inappropriate.

Assessment of reporting bias

Publication bias will be assessed, when possible, using a funnel plot of the studies' standard error or precision as a function of effect estimates.

Assessment of heterogeneity

The variability across studies (for each outcome) will be assessed using the I^2 statistic. Percentages of 0–40%, 41–60% or 61–100% respectively indicate low, medium or high heterogeneity. In addition, a chi-squared test will be performed to assess the statistical significance of the heterogeneity.

Dealing with missing data

Missing data will be obtained by contacting the corresponding authors, where possible. Missing SDs and correlation coefficients will be estimated or imputed from the other available data using protocols explained in the Cochrane Handbook.

For peer review only - http://bmjopen.bmj.com/site/about/guidelines.xhtml

Subgroup analysis

Plausible sources of heterogeneity will be explored using a subgroup analysis of the participant's age, degree of hearing loss, level of experience with hearing aids and the length of their acclimatisation period with the hearing aid.

Confidence in the cumulative estimate

The Grading of Recommendations, Assessment, Development and Evaluations tool (GRADE) will be used to rate the overall evidence of each outcome. The assigned rating (i.e. high, moderate, low or very low) reflects our confidence of the pooled estimate, that is, high-quality evidence implies that the pooled estimates are probably very close to the true effect of the intervention. Conversely, very low-quality evidence implies that the pooled estimate is likely to be substantially different from the true effect and further research is needed to strengthen our confidence in the pooled effect. The initial quality ratings for randomised and non-randomised trials will be respectively high and low. Cross-over designs, where each participant acts as their own control, will also initially be assigned a high quality provided cross-over order is counterbalanced across participants. The assigned rating can be upgraded or downgraded by one or two points based on the seriousness of multiple factors. Notably, three factors may upgrade the quality of evidence: large effect size, dose-response gradient and plausible confounding factors that likely have reduced the effect observed. Meanwhile, study limitations, inconsistency, indirectness, imprecision and publication bias are factors that may reduce our confidence in the pooled estimates. The overall quality rating for each outcome will be presented in a summary of findings table utilizing the GRADEpro online tool (https://gradepro.org/). The review team will independently grade the overall quality of each outcome. Disagreements will be resolved through discussion or consensus.

Sensitivity analysis

The impact of imputing missing data, potential confounders and studies with high risk of bias on the robustness of the findings will be assessed using a sensitivity analysis, that is, the meta-analysis will be performed twice, with and without such studies.

Patient and Public Involvement

The representatives of patients and public involvement groups were not involved in the development of this review protocol. The review findings will be disseminated to the public through social media and other public platforms.

For peer review only - http://bmjopen.bmj.com/site/about/guidelines.xhtml

ETHICS AND DISSEMINATION

We will only retrieve and analyse data from existing studies, hence no ethical approval is required. The review findings will be published in a peer-reviewed journal and presented in scientific conferences.

Contributor.

IA, the guarantor of this review, developed and drafted the initial version of this protocol and tested the preliminary search strategy. KJM and HD contributed to the development, critically reviewed and provided extensive feedback on the earlier versions of this manuscript. Jan Schoones (an information specialist, Systematic Review Solutions Limited) searched the databases. All authors approved this version of the manuscript.

Funding NIHR Manchester Biomedical Research Centre (funder reference: IS-BRC-1215-20007)

Competing interest None declared.

ACKNOWLEDGEMENTS

IA, KJM and HD are supported by the NIHR Manchester Biomedical research Centre. The first author is also supported by the Deanship of Scientific Research at the College of Applied Medical Sciences Research Center at King Saud University.

For peer review only - http://bmjopen.bmj.com/site/about/guidelines.xhtml

Reference

1. World Health Organization. *Prevention of blindness and deafness*. 2018 [cited 2019 06/10/2019]; Available from: https://www.who.int/pbd/deafness/estimates/en/.
2. World Health Organization, *Global Health Estimates 2016: Disease burden by Cause, Age, Sex, by Country and by Region, 2000-2016*. 2018: Geneva.
3. Davis, A., et al., *Acceptability, benefit and costs of early screening for hearing disability: a study of potential screening tests and models.* Health Technol Assess, 2007. **11**(42): p. 1-294.
4. Thyer, N.J., et al., *Randomised controlled feasibility trial of the Active Communication Education programme plus hearing aid provision versus hearing aid provision alone (ACE to HEAR): a study protocol.* BMJ Open, 2018. **8**(7): p. e021502.
5. Moore, B.C. and G. Popelka, *Introduction to Hearing Aid*, in *Hearing Aids*, G.R. Popelka, et al., Editors. 2016, Springer: Switzerland.
6. Kochkin, S., *MarkeTrak VIII: 25-year trends in the hearing health market.* Hearing Review, 2009. **16**(11): p. 12-31.
7. Ferguson, M.A., et al., *Hearing aids for mild to moderate hearing loss in adults.* Cochrane Database Syst Rev, 2017. **9**: p. CD012023.
8. Munro, K. and G.H. Mueller, *Clinical Verification of Hearing Aid Performance*, in *Hearing Aids*, G. Popelka, et al., Editors. 2016, Springer Switzeland.
9. Nance, G. and D. Causey, *HEARING-AID EVALUATION: AN EXAMINATION OF TWO PROCEDURES.* Bulletin of Prosthetics Research - Spring, 1968.
10. Jones, I.H. and V.O. Knudsen, *What audiometry can now mean in routine practice.* Journal of the American Medical Association, 1938. **111**(7): p. 597-605.
11. Watson, N.A. and V.O. Knudsen, *Selective Amplification in Hearing Aids.* The Journal of the Acoustical Society of America, 1940. **11**(4): p. 406-419.
12. Lybarger, S., *US patent application SN543-278.* 1944.
13. Dillon, H. *The Research of Denis Byrne at NAL: Implications for Clinicians Today*. 2001 [cited 2020 25/04]; Available from: https://www.audiologyonline.com/articles/research-denis-byrne-at-nal-1200.
14. Byrne, D., et al., *NAL-NL1 procedure for fitting nonlinear hearing aids: characteristics and comparisons with other procedures.* J Am Acad Audiol, 2001. **12**(1): p. 37-51.
15. Keidser, G., et al., *The NAL-NL2 Prescription Procedure.* Audiol Res, 2011. **1**(1): p. e24.
16. Scollie, S., et al., *The Desired Sensation Level multistage input/output algorithm.* Trends Amplif, 2005. **9**(4): p. 159-97.
17. Dillon, H., *Hearing Aids*. 2012: Thieme.
18. Keidser, G., et al., *NAL-NL2 empirical adjustments.* Trends Amplif, 2012. **16**(4): p. 211-23.
19. Almufarrij, I., et al., *Direct-to-Consumer Hearing Devices: Capabilities, Costs, and Cosmetics.* Trends Hear, 2019. **23**: p. 2331216519858301.
20. Sabin, A.T., et al., *Validation of a Self-Fitting Method for Over-the-Counter Hearing Aids.* Trends Hear, 2020. **24**: p. 2331216519900589.
21. Scollie, S., et al., *Evaluation of the NAL-NL1 and DSL v4.1 prescriptions for children: Preference in real world use.* Int J Audiol, 2010. **49 Suppl 1**: p. S49-63.
22. Walravens, E., G. Keidser, and L. Hickson, *Consistency of Hearing Aid Setting Preference in Simulated Real-World Environments: Implications for Trainable Hearing Aids.* Trends Hear, 2020. **24**: p. 2331216520933392.
23. Mueller, H.G., *Fitting hearing aids to adults using prescriptive methods: an evidence-based review of effectiveness.* J Am Acad Audiol, 2005. **16**(7): p. 448-60.
24. Shamseer, L., et al., *Preferred reporting items for systematic review and meta-analysis protocols (PRISMA-P) 2015: elaboration and explanation.* Bmj, 2015. **350**: p. g7647.
25. Ventry, I.M. and B.E. Weinstein, *The hearing handicap inventory for the elderly: a new tool.* Ear Hear, 1982. **3**(3): p. 128-34.

For peer review only - http://bmjopen.bmj.com/site/about/guidelines.xhtml

26. Gatehouse, S. and W. Noble, *The Speech, Spatial and Qualities of Hearing Scale (SSQ).* International journal of audiology, 2004. **43**(2): p. 85-99.

For peer review only

For peer review only - http://bmjopen.bmj.com/site/about/guidelines.xhtml

Appendix 1: search protocols

PubMed:

(("Hearing Loss"[Mesh] OR "hearing loss"[tw] OR "hearing losses"[tw] OR "Hearing Disorders"[Mesh] OR "hearing disorder"[tw] OR "hearing disorders"[tw] OR "hearing problem"[tw] OR "hearing problems"[tw] OR "hearing impaired"[tw] OR "hearing difficulties"[tw] OR "hearing difficulty"[tw] OR "ANSD"[tw] OR "auditory neuropathy"[tw]) AND ("Hearing Aids"[Mesh] OR "hearing aid"[tw] OR "hearing aids"[tw] OR "hearing device"[tw] OR "hearing devices"[tw] OR "acoustic aid"[tw] OR "acoustic aids"[tw] OR " hearing instrument"[tw] OR " hearing instruments"[tw] OR "over-the-counter hearing"[tw] OR "over the counter hearing "[tw] OR "direct-to-consumer hearing"[tw] OR "direct to consumer hearing"[tw] OR "self-fitting hearing"[tw] OR "self fitting hearing "[tw] OR "personal sound amplification"[tw]) AND ("real ear measurement"[tw] OR "real ear measurements"[tw] OR "real ear measure"[tw] OR "real ear measures"[tw] OR "real-ear measurement"[tw] OR "real-ear measurements"[tw] OR "real-ear measure"[tw] OR "real-ear measures"[tw] OR "REM"[tw] OR "REMs"[tw] OR "PMM"[tw] OR "PMMs"[tw] OR "probe microphone"[tw] OR "probe microphones"[tw] OR "probe mic"[tw] OR "probe mics"[tw] OR "probe microphone measurement"[tw] OR "probe microphone measurements"[tw] OR "probe microphone measure"[tw] OR "probe microphone measures"[tw] OR "probemicrophone"[tw] OR "probe-microphones"[tw] OR "probe-microphone measurement"[tw] OR "probe-microphone measurements"[tw] OR "probe-microphone measure"[tw] OR "probe-microphone measures"[tw] OR "real-ear verification"[tw] OR "audiology best practices"[tw] OR "audiology best practice"[tw] OR "best practices"[tw] OR "best practice"[tw] OR "clinician-driven"[tw] OR "physician-driven"[tw] OR "doctor-driven"[tw] OR "prescriptive methods"[tw] OR "prescription methods"[tw] OR "prescriptive method*"[tw] OR "prescription method*"[tw] OR "formula"[tw] OR "formulae"[tw]))

MEDLINE (OVID)

((exp "Hearing Loss"/ OR "hearing loss".mp OR "hearing losses".mp OR exp "Hearing Disorders"/ OR "hearing disorder".mp OR "hearing disorders".mp OR "hearing problem".mp OR "hearing problems".mp OR "hearing impaired".mp OR "hearing difficulties".mp OR "hearing difficulty".mp OR "ANSD".mp OR "auditory neuropathy".mp) AND (exp "Hearing Aids"/ OR "hearing aid".mp OR "hearing aids".mp OR "hearing device".mp OR "hearing devices".mp OR "acoustic aid".mp OR "acoustic aids".mp OR " hearing instrument".mp OR " hearing instruments".mp OR "over-the-counter hearing".mp OR "over the counter hearing ".mp OR "direct-to-consumer hearing".mp OR "direct to consumer hearing".mp OR "self-fitting hearing".mp OR "self fitting hearing ".mp OR "personal sound amplification".mp) AND ("real ear measurement".mp OR "real ear measurements".mp OR "real ear measure".mp OR "real ear measures".mp OR "real-ear measurement".mp OR "real-ear measurements".mp OR "real-ear measure".mp OR "real-ear measures".mp OR "REM".mp OR "REMs".mp OR "PMM".mp OR "PMMs".mp OR "probe microphone".mp OR "probe microphones".mp OR "probe mic".mp OR "probe mics".mp OR "probe microphone measurement".mp OR "probe microphone measurements".mp OR "probe microphone measure".mp OR "probe microphone measures".mp OR "probemicrophone".mp OR "probe-microphones".mp OR "probe-microphone measurement".mp OR "probe-microphone measurements".mp OR "probe-microphone measure".mp OR "probe-microphone measures".mp OR "real-ear verification".mp OR "audiology best practices".mp OR "audiology best practice".mp OR "best practices".mp OR "best practice".mp OR "clinician-driven".mp OR "physician-driven".mp OR "doctor-driven".mp OR "prescriptive methods".mp OR "prescription methods".mp OR "prescriptive method*".mp OR "prescription method*".mp OR "formula".mp OR "formulae".mp))

For peer review only

Embase (OVID)

((exp "Hearing Impairment"/ OR "hearing loss".mp OR "hearing losses".mp OR exp "Hearing Disorder"/ OR "hearing disorder".mp OR "hearing disorders".mp OR "hearing problem".mp OR "hearing problems".mp OR "hearing impaired".mp OR "hearing difficulties".mp OR "hearing difficulty".mp OR "ANSD".mp OR "auditory neuropathy".mp) AND (exp "Hearing Aid"/ OR "hearing aid".mp OR "hearing aids".mp OR "hearing device".mp OR "hearing devices".mp OR "acoustic aid".mp OR "acoustic aids".mp OR " hearing instrument".mp OR " hearing instruments".mp OR "over-the-counter hearing".mp OR "over the counter hearing ".mp OR "direct-to-consumer hearing".mp OR "direct to consumer hearing".mp OR "self-fitting hearing".mp OR "self fitting hearing ".mp OR "personal sound amplification".mp) AND ("real ear measurement".mp OR "real ear measurements".mp OR "real ear measure".mp OR "real ear measures".mp OR "real-ear measurement".mp OR "real-ear measurements".mp OR "real-ear measure".mp OR "real-ear measures".mp OR "REM".mp OR "REMs".mp OR "PMM".mp OR "PMMs".mp OR "probe microphone".mp OR "probe microphones".mp OR "probe mic".mp OR "probe mics".mp OR "probe microphone measurement".mp OR "probe microphone measurements".mp OR "probe microphone measure".mp OR "probe microphone measures".mp OR "probemicrophone".mp OR "probe-microphones".mp OR "probe-microphone measurement".mp OR "probe-microphone measurements".mp OR "probe-microphone measure".mp OR "probe-microphone measures".mp OR "real-ear verification".mp OR "audiology best practices".mp OR "audiology best practice".mp OR "best practices".mp OR "best practice".mp OR "clinician-driven".mp OR "physician-driven".mp OR "doctor-driven".mp OR "prescriptive methods".mp OR "prescription methods".mp OR "prescriptive method*".mp OR "prescription method*".mp OR "formula".mp OR "formulae".mp))

PsycINFO

TX(("Hearing Impairment" OR "hearing loss" OR "hearing losses" OR "Hearing Disorder" OR "hearing disorder" OR "hearing disorders" OR "hearing problem" OR "hearing problems" OR "hearing impaired" OR "hearing difficulties" OR "hearing difficulty" OR "ANSD" OR "auditory neuropathy") AND ("Hearing Aid" OR "hearing aid" OR "hearing aids" OR "hearing device" OR "hearing devices" OR "acoustic aid" OR "acoustic aids" OR " hearing instrument" OR " hearing instruments" OR "over-the-counter hearing" OR "over the counter hearing " OR "direct-to-consumer hearing" OR "direct to consumer hearing" OR "self-fitting hearing" OR "self fitting hearing " OR "personal sound amplification") AND ("real ear measurement" OR "real ear measurements" OR "real ear measure" OR "real ear measures" OR "real-ear measurement" OR "real-ear measurements" OR "real-ear measure" OR "real-ear measures" OR "REM" OR "REMs" OR "PMM" OR "PMMs" OR "probe microphone" OR "probe microphones" OR "probe mic" OR "probe mics" OR "probe microphone measurement" OR "probe microphone measurements" OR "probe microphone measure" OR "probe microphone measures" OR "probemicrophone" OR "probe-microphones" OR "probe-microphone measurement" OR "probe-microphone measurements" OR "probe-microphone measure" OR "probe-microphone measures" OR "real-ear verification" OR "audiology best practices" OR "audiology best practice" OR "best practices" OR "best practice" OR "clinician-driven" OR "physician-driven" OR "doctor-driven" OR "prescriptive methods" OR "prescription methods" OR "prescriptive method*" OR "prescription method*" OR "formula" OR "formulae"))

Web of Science (all databases)

TS=(("Hearing Impairment" OR "hearing loss" OR "hearing losses" OR "Hearing Disorder" OR "hearing disorder" OR "hearing disorders" OR "hearing problem" OR "hearing problems" OR "hearing impaired" OR "hearing difficulties" OR "hearing difficulty" OR "ANSD" OR "auditory neuropathy") AND ("Hearing Aid" OR "hearing aid" OR "hearing aids" OR "hearing device" OR "hearing devices" OR "acoustic aid" OR "acoustic aids" OR " hearing instrument" OR " hearing instruments" OR "over-the-counter hearing" OR "over the counter hearing " OR "direct-to-consumer hearing" OR "direct to consumer hearing" OR "self-fitting hearing" OR "self fitting hearing " OR "personal sound amplification") AND ("real ear measurement" OR "real ear measurements" OR "real ear measure" OR "real ear measures" OR "real-ear measurement" OR "real-ear measurements" OR "real-ear measure" OR "real-ear measures" OR "REM" OR "REMs" OR "PMM" OR "PMMs" OR "probe microphone" OR "probe microphones" OR "probe mic" OR "probe mics" OR "probe microphone measurement" OR "probe microphone measurements" OR "probe microphone measure" OR "probe microphone measures" OR "probemicrophone" OR "probe-microphones" OR "probe-microphone measurement" OR "probe-microphone measurements" OR "probe-microphone measure" OR "probe-microphone measures" OR "real-ear verification" OR "audiology best practices" OR "audiology best practice" OR "best practices" OR "best practice" OR "clinician-driven" OR "physician-driven" OR "doctor-driven" OR "prescriptive methods" OR "prescription methods" OR "prescriptive method*" OR "prescription method*" OR "formula" OR "formulae"))

Cochrane Library

(("Hearing Impairment" OR "hearing loss" OR "hearing losses" OR "Hearing Disorder" OR "hearing disorder" OR "hearing disorders" OR "hearing problem" OR "hearing problems" OR "hearing impaired" OR "hearing difficulties" OR "hearing difficulty" OR "ANSD" OR "auditory neuropathy") AND ("Hearing Aid" OR "hearing aid" OR "hearing aids" OR "hearing device" OR "hearing devices" OR "acoustic aid" OR "acoustic aids" OR " hearing instrument" OR " hearing instruments" OR "over-the-counter hearing" OR "over the counter hearing " OR "direct-to-consumer hearing" OR "direct to consumer hearing" OR "self-fitting hearing" OR "self fitting hearing " OR "personal sound amplification") AND ("real ear measurement" OR "real ear measurements" OR "real ear measure" OR "real ear measures" OR "real-ear measurement" OR "real-ear measurements" OR "real-ear measure" OR "real-ear measures" OR "REM" OR "REMs" OR "PMM" OR "PMMs" OR "probe microphone" OR "probe microphones" OR "probe mic" OR "probe mics" OR "probe microphone measurement" OR "probe microphone measurements" OR "probe microphone measure" OR "probe microphone measures" OR "probemicrophone" OR "probe-microphones" OR "probe-microphone measurement" OR "probe-microphone measurements" OR "probe-microphone measure" OR "probe-microphone measures" OR "real-ear verification" OR "audiology best practices" OR "audiology best practice" OR "best practices" OR "best practice" OR "clinician-driven" OR "physician-driven" OR "doctor-driven" OR "prescriptive methods" OR "prescription methods" OR "prescriptive method*" OR "prescription method*" OR "formula" OR "formulae")):ti,ab,kw

Emcare

((exp "Hearing Impairment"/ OR "hearing loss".mp OR "hearing losses".mp OR exp "Hearing Disorder"/ OR "hearing disorder".mp OR "hearing disorders".mp OR "hearing problem".mp OR "hearing problems".mp OR "hearing impaired".mp OR "hearing difficulties".mp OR "hearing difficulty".mp OR "ANSD".mp OR "auditory neuropathy".mp) AND (exp "Hearing Aid"/ OR "hearing aid".mp OR "hearing aids".mp OR "hearing device".mp OR "hearing devices".mp OR

For peer review only

"acoustic aid".mp OR "acoustic aids".mp OR " hearing instrument".mp OR " hearing instruments".mp OR "over-the-counter hearing".mp OR "over the counter hearing ".mp OR "direct-to-consumer hearing".mp OR "direct to consumer hearing".mp OR "self-fitting hearing".mp OR "self fitting hearing ".mp OR "personal sound amplification".mp) AND ("real ear measurement".mp OR "real ear measurements".mp OR "real ear measure".mp OR "real ear measures".mp OR "real-ear measurement".mp OR "real-ear measurements".mp OR "real-ear measure".mp OR "real-ear measures".mp OR "REM".mp OR "REMs".mp OR "PMM".mp OR "PMMs".mp OR "probe microphone".mp OR "probe microphones".mp OR "probe mic".mp OR "probe mics".mp OR "probe microphone measurement".mp OR "probe microphone measurements".mp OR "probe microphone measure".mp OR "probe microphone measures".mp OR "probemicrophone".mp OR "probe-microphones".mp OR "probe-microphone measurement".mp OR "probe-microphone measurements".mp OR "probe-microphone measure".mp OR "probe-microphone measures".mp OR "real-ear verification".mp OR "audiology best practices".mp OR "audiology best practice".mp OR "best practices".mp OR "best practice".mp OR "clinician-driven".mp OR "physician-driven".mp OR "doctor-driven".mp OR "prescriptive methods".mp OR "prescription methods".mp OR "prescriptive method*".mp OR "prescription method*".mp OR "formula".mp OR "formulae".mp))

Academic Search Premier

TI(("Hearing Impairment" OR "hearing loss" OR "hearing losses" OR "Hearing Disorder" OR "hearing disorder" OR "hearing disorders" OR "hearing problem" OR "hearing problems" OR "hearing impaired" OR "hearing difficulties" OR "hearing difficulty" OR "ANSD" OR "auditory neuropathy") AND ("Hearing Aid" OR "hearing aid" OR "hearing aids" OR "hearing device" OR "hearing devices" OR "acoustic aid" OR "acoustic aids" OR " hearing instrument" OR " hearing instruments" OR "over-the-counter hearing" OR "over the counter hearing " OR "direct-to-consumer hearing" OR "direct to consumer hearing" OR "self-fitting hearing" OR "self fitting hearing " OR "personal sound amplification") AND ("real ear measurement" OR "real ear measurements" OR "real ear measure" OR "real ear measures" OR "real-ear measurement" OR "real-ear measurements" OR "real-ear measure" OR "real-ear measures" OR "REM" OR "REMs" OR "PMM" OR "PMMs" OR "probe microphone" OR "probe microphones" OR "probe mic" OR "probe mics" OR "probe microphone measurement" OR "probe microphone measurements" OR "probe microphone measure" OR "probe microphone measures" OR "probemicrophone" OR "probe-microphones" OR "probe-microphone measurement" OR "probe-microphone measurements" OR "probe-microphone measure" OR "probe-microphone measures" OR "real-ear verification" OR "audiology best practices" OR "audiology best practice" OR "best practices" OR "best practice" OR "clinician-driven" OR "physician-driven" OR "doctor-driven" OR "prescriptive methods" OR "prescription methods" OR "prescriptive method*" OR "prescription method*" OR "formula" OR "formulae")) OR SU(("Hearing Impairment" OR "hearing loss" OR "hearing losses" OR "Hearing Disorder" OR "hearing disorder" OR "hearing disorders" OR "hearing problem" OR "hearing problems" OR "hearing impaired" OR "hearing difficulties" OR "hearing difficulty" OR "ANSD" OR "auditory neuropathy") AND ("Hearing Aid" OR "hearing aid" OR "hearing aids" OR "hearing device" OR "hearing devices" OR "acoustic aid" OR "acoustic aids" OR " hearing instrument" OR " hearing instruments" OR "over-the-counter hearing" OR "over the counter hearing " OR "direct-to-consumer hearing" OR "direct to consumer hearing" OR "self-fitting hearing" OR "self fitting hearing " OR "personal sound amplification") AND ("real ear measurement" OR "real ear measurements" OR "real ear measure" OR "real ear measures" OR "real-ear measurement" OR "real-ear measurements" OR "real-ear measure" OR "real-ear measures" OR "REM" OR "REMs" OR "PMM" OR "PMMs" OR "probe microphone" OR "probe microphones" OR "probe mic" OR "probe mics" OR "probe microphone measurement" OR "probe microphone measurements" OR "probe microphone measure" OR "probe microphone

For peer review only

measures" OR "probemicrophone" OR "probe-microphones" OR "probe-microphone measurement" OR "probe-microphone measurements" OR "probe-microphone measure" OR "probe-microphone measures" OR "real-ear verification" OR "audiology best practices" OR "audiology best practice" OR "best practices" OR "best practice" OR "clinician-driven" OR "physician-driven" OR "doctor-driven" OR "prescriptive methods" OR "prescription methods" OR "prescriptive method*" OR "prescription method*" OR "formula" OR "formulae")) OR KW(("Hearing Impairment" OR "hearing loss" OR "hearing losses" OR "Hearing Disorder" OR "hearing disorder" OR "hearing disorders" OR "hearing problem" OR "hearing problems" OR "hearing impaired" OR "hearing difficulties" OR "hearing difficulty" OR "ANSD" OR "auditory neuropathy") AND ("Hearing Aid" OR "hearing aid" OR "hearing aids" OR "hearing device" OR "hearing devices" OR "acoustic aid" OR "acoustic aids" OR " hearing instrument" OR " hearing instruments" OR "over-the-counter hearing" OR "over the counter hearing " OR "direct-to-consumer hearing" OR "direct to consumer hearing" OR "self-fitting hearing" OR "self fitting hearing " OR "personal sound amplification") AND ("real ear measurement" OR "real ear measurements" OR "real ear measure" OR "real ear measures" OR "real-ear measurement" OR "real-ear measurements" OR "real-ear measure" OR "real-ear measures" OR "REM" OR "REMs" OR "PMM" OR "PMMs" OR "probe microphone" OR "probe microphones" OR "probe mic" OR "probe mics" OR "probe microphone measurement" OR "probe microphone measurements" OR "probe microphone measure" OR "probe microphone measures" OR "probemicrophone" OR "probe-microphones" OR "probe-microphone measurement" OR "probe-microphone measurements" OR "probe-microphone measure" OR "probe-microphone measures" OR "real-ear verification" OR "audiology best practices" OR "audiology best practice" OR "best practices" OR "best practice" OR "clinician-driven" OR "physician-driven" OR "doctor-driven" OR "prescriptive methods" OR "prescription methods" OR "prescriptive method*" OR "prescription method*" OR "formula" OR "formulae")) OR AB(("Hearing Impairment" OR "hearing loss" OR "hearing losses" OR "Hearing Disorder" OR "hearing disorder" OR "hearing disorders" OR "hearing problem" OR "hearing problems" OR "hearing impaired" OR "hearing difficulties" OR "hearing difficulty" OR "ANSD" OR "auditory neuropathy") AND ("Hearing Aid" OR "hearing aid" OR "hearing aids" OR "hearing device" OR "hearing devices" OR "acoustic aid" OR "acoustic aids" OR " hearing instrument" OR " hearing instruments" OR "over-the-counter hearing" OR "over the counter hearing " OR "direct-to-consumer hearing" OR "direct to consumer hearing" OR "self-fitting hearing" OR "self fitting hearing " OR "personal sound amplification") AND ("real ear measurement" OR "real ear measurements" OR "real ear measure" OR "real ear measures" OR "real-ear measurement" OR "real-ear measurements" OR "real-ear measure" OR "real-ear measures" OR "REM" OR "REMs" OR "PMM" OR "PMMs" OR "probe microphone" OR "probe microphones" OR "probe mic" OR "probe mics" OR "probe microphone measurement" OR "probe microphone measurements" OR "probe microphone measure" OR "probe microphone measures" OR "probemicrophone" OR "probe-microphones" OR "probe-microphone measurement" OR "probe-microphone measurements" OR "probe-microphone measure" OR "probe-microphone measures" OR "real-ear verification" OR "audiology best practices" OR "audiology best practice" OR "best practices" OR "best practice" OR "clinician-driven" OR "physician-driven" OR "doctor-driven" OR "prescriptive methods" OR "prescription methods" OR "prescriptive method*" OR "prescription method*" OR "formula" OR "formulae"))

For peer review only

Appendix 2: Data items

General information	Method	Participants	Intervention	Outcome	Funding source	Declaration of interest	Additional comments
Title<br>Authors<br>Publication year	Study design<br>Conditions and the full study durations<br>Sequence generation<br>Sequence concealment<br>Blinding<br>Power analysis	Sample size<br>Country / Setting<br>Age<br>Sex<br>Inclusion and exclusion criteria	Intervention group<br>Comparator group	Primary outcome<br>Secondary outcome(s)<br>Adverse events			

For peer review only

Preferred reporting items for systematic review and meta-analysis protocols (PRISMA-P) 2015

PRISMA-P 2015 Checklist

Section/topic	Item No	Checklist item	Information reported Yes	No	Page number
**ADMINISTRATIVE INFORMATION**					
**Title**					
Identification	1a	Identify the report as a protocol of a systematic review	X		1
Update	1b	If the protocol is for an update of a previous systematic review, identify as such		X	Not an update
**Registration**	2	If registered, provide the name of the registry (e.g., PROSPERO) and registration number in the Abstract	X		2
**Authors**					
Contact	3a	Provide name, institutional affiliation, and e-mail address of all protocol authors; provide physical mailing address of corresponding author	X		1
Contributions	3b	Describe contributions of protocol authors and identify the guarantor of the review	X		10
**Amendments**	4	If the protocol represents an amendment of a previously completed or published protocol, identify as such and list changes; otherwise, state plan for documenting important protocol amendments		X	Not an amendment
**Support**					
Sources	5a	Indicate sources of financial or other support for the review	X		10
Sponsor	5b	Provide name for the review funder and/or sponsor	X		10
Role of sponsor/funder	5c	Describe roles of funder(s), sponsor(s), and/or institution(s), if any, in developing the protocol	X		10
**INTRODUCTION**					
**Rationale**	6	Describe the rationale for the review in the context of what is already known	X		4
**Objectives**	7	Provide an explicit statement of the question(s) the review will address with reference to participants, interventions, comparators, and outcomes (PICO)	X		4-5
**METHODS**					

For peer review only

**Eligibility criteria**	8	Specify the study characteristics (e.g., PICO, study design, setting, time frame) and report characteristics (e.g., years considered, language, publication status) to be used as criteria for eligibility for the review	☒	☐	5-6
**Information sources**	9	Describe all intended information sources (e.g., electronic databases, contact with study authors, trial registers, or other grey literature sources) with planned dates of coverage	☒	☐	6
**Search strategy**	10	Present draft of search strategy to be used for at least one electronic database, including planned limits, such that it could be repeated	☒	☐	Appendix 1
STUDY RECORDS					
Data management	11a	Describe the mechanism(s) that will be used to manage records and data throughout the review	☒	☐	6-7
Selection process	11b	State the process that will be used for selecting studies (e.g., two independent reviewers) through each phase of the review (i.e., screening, eligibility, and inclusion in meta-analysis)	☒	☐	7
Data collection process	11c	Describe planned method of extracting data from reports (e.g., piloting forms, done independently, in duplicate), any processes for obtaining and confirming data from investigators	☒	☐	7
**Data items**	12	List and define all variables for which data will be sought (e.g., PICO items, funding sources), any pre-planned data assumptions and simplifications	☒	☐	7 and Appendix 2
**Outcomes and prioritization**	13	List and define all outcomes for which data will be sought, including prioritization of main and additional outcomes, with rationale	☒	☐	6
**Risk of bias in individual studies**	14	Describe anticipated methods for assessing risk of bias of individual studies, including whether this will be done at the outcome or study level, or both; state how this information will be used in data synthesis	☒	☐	7
DATA					
**Synthesis**	15a	Describe criteria under which study data will be quantitatively synthesized	☒	☐	8
	15b	If data are appropriate for quantitative synthesis, describe planned summary measures, methods of handling data, and methods of combining data from studies, including any planned exploration of consistency (e.g., I^2, Kendall's tau)	☒	☐	8
	15c	Describe any proposed additional analyses (e.g., sensitivity or subgroup analyses, meta-regression)	☒	☐	9
	15d	If quantitative synthesis is not appropriate, describe the type of summary planned	☒	☐	8
**Meta-bias(es)**	16	Specify any planned assessment of meta-bias(es) (e.g., publication bias across studies, selective reporting within studies)	☒	☐	8-9
**Confidence in cumulative evidence**	17	Describe how the strength of the body of evidence will be assessed (e.g., GRADE)	☒	☐	9

For peer review only

BMJ Open

Does an individually applied audiogram-based adult prescription formula affect outcome? A systematic review protocol.

Journal:	*BMJ Open*
Manuscript ID	bmjopen-2020-045899.R1
Article Type:	Protocol
Date Submitted by the Author:	18-Mar-2021
Complete List of Authors:	Almufarrij, Ibrahim; The University of Manchester, Manchester Centre for Audiology and Deafness, School of Health Sciences; King Saud University, Department of Rehabilitation Sciences<br>Dillon, Harvey; The University of Manchester, Manchester Centre for Audiology and Deafness, School of Health Sciences; Macquarie University, Department of Linguistics<br>Munro, Kevin; The University of Manchester, Manchester Centre for Audiology and Deafness, School of Health Sciences; Manchester University NHS Foundation Trust, Manchester University Hospitals NHS Foundation Trust, Manchester Academic Health Science Centre,
**Primary Subject Heading**:	Ear, nose and throat/otolaryngology
Secondary Subject Heading:	Communication, Ear, nose and throat/otolaryngology, Evidence based practice
Keywords:	Audiology < OTOLARYNGOLOGY, Neurotology < OTOLARYNGOLOGY, Adult otolaryngology < OTOLARYNGOLOGY

SCHOLARONE™
Manuscripts

I, the Submitting Author has the right to grant and does grant on behalf of all authors of the Work (as defined in the below author licence), an exclusive licence and/or a non-exclusive licence for contributions from authors who are: i) UK Crown employees; ii) where BMJ has agreed a CC-BY licence shall apply, and/or iii) in accordance with the terms applicable for US Federal Government officers or employees acting as part of their official duties; on a worldwide, perpetual, irrevocable, royalty-free basis to BMJ Publishing Group Ltd ("BMJ") its licensees and where the relevant Journal is co-owned by BMJ to the co-owners of the Journal, to publish the Work in this journal and any other BMJ products and to exploit all rights, as set out in our licence.

The Submitting Author accepts and understands that any supply made under these terms is made by BMJ to the Submitting Author unless you are acting as an employee on behalf of your employer or a postgraduate student of an affiliated institution which is paying any applicable article publishing charge ("APC") for Open Access articles. Where the Submitting Author wishes to make the Work available on an Open Access basis (and intends to pay the relevant APC), the terms of reuse of such Open Access shall be governed by a Creative Commons licence – details of these licences and which Creative Commons licence will apply to this Work are set out in our licence referred to above.

Other than as permitted in any relevant BMJ Author's Self Archiving Policies, I confirm this Work has not been accepted for publication elsewhere, is not being considered for publication elsewhere and does not duplicate material already published. I confirm all authors consent to publication of this Work and authorise the granting of this licence.

For peer review only

Does an individually applied audiogram-based adult prescription formula affect outcome? A systematic review protocol.

Authors: Ibrahim Almufarrij[1, 2], Harvey Dillon[1,4] and Kevin J Munro[1, 3]

Author affiliations

[1] Manchester Centre for Audiology and Deafness, School of Health Sciences, University of Manchester and the Manchester Academic Health Science Centre, UK

[2] Department of Rehabilitation Sciences, College of Applied Medical Sciences, King Saud University, Riyadh, Kingdom of Saudi Arabia

[3] Manchester University Hospitals NHS Foundation Trust, Manchester Academic Health Science Centre, UK

[4] Department of Linguistics, Macquarie University, Sydney, Australia

Corresponding author: Ibrahim Almufarrij, Manchester Centre for Audiology and Deafness, University of Manchester, Manchester, M13 9PL, United Kingdom. E-mail: Email: ialmufarrij@ksu.edu.sa

For peer review only

ABSTRACT

Introduction: Hearing aids are typically programmed using the individual's audiometric thresholds. Developments in technology have resulted in a new category of direct-to-consumer devices, which are not programmed using the individual's audiometric thresholds. This review aims to identify whether programming hearing aids using the individual's audiogram-based prescription results in better outcomes for adults with hearing loss.

Methods and analysis: The methods of this review are reported in line with Preferred Reporting Items for Systematic Reviews and Meta-Analyses Protocols guidelines. On 23 August 2020, eight different databases were systematically searched without any restrictions: EMBASE, MEDLINE, PubMed, PsycINFO, Web of Science, Cochrane Library, Emcare and Academic Search Premier. To ensure that this review includes the most recent evidence, the searches will be repeated at the final write-up stage. The population of interest of this review will be adults with any degree or type of hearing loss. The studies should compare hearing aids programmed using an audiogram-based prescription (and verified in the real ear) to those not programmed on the basis of the individual's audiogram. The primary outcome of interest is consumers' listening preferences. Hearing-specific health-related quality of life, self-reported listening ability, speech intelligibility of words and sentences in quiet and noisy situations, sound quality ratings and adverse events are the secondary outcomes of interest. Both randomised and non-randomised controlled trials will be included. The quality of each individual study and the overall evidence will be assessed using Down and Black's checklist and the Grading of Recommendations, Assessment, Development and Evaluations tool, respectively.

Ethics and dissemination: We will only retrieve and analyse data from published studies, so no ethical approval is required. The review findings will be published in a peer-reviewed journal and presented at scientific conferences.

PROSPERO registration number: CRD42020197232

For peer review only

Strengths and limitations of the study:

- The primary outcome is listening preference, measured in a within-group (cross-over) experiment, but both within- and between-group design studies will be included as the latter design can contribute to secondary outcomes.
- Amplification devices pre-programmed for generic mild or moderate hearing loss will be included amongst the non-individually programmed devices.
- We will only include studies that compare programming approaches using the same hearing device.
- Magazine articles, conference abstracts, clinical guidelines and theses will be excluded.
- The results will help decision makers and hearing health professionals to provide better patient-centred care.

INTRODUCTION

Hearing loss is a debilitating health condition that affects more than 450 million individuals worldwide [1]. It is also the most common cause for years lived with disability [2]. Hearing loss can directly affect people's ability to communicate, which can consequently lead to depression and poor social interaction [3]. It is also associated with a reduced quality of life and an increased risk of dementia [4]. Hearing loss can be partially and successfully mitigated through the use of complex electroacoustic amplification devices known as hearing aids [5].

Hearing aids are the primary and the most common clinical intervention for hearing loss because they are very effective in improving hearing-related quality of life [6, 7]. The ultimate goals for hearing aids are to restore the audibility of soft level sounds, maximise the intelligibility of conversational-level sounds and ensure that loud sounds are within comfortable levels [8].

In the early 1940s and 1950s, the most controversial aspects of clinical audiology were hearing aid selection and fitting, as experts could not agree upon the most appropriate approaches [9]. Since this period, prescriptive approaches have evolved to provide recommended gains for each audiometric frequency and input level. The concept of hearing aid prescriptions goes back to the early 1940s when Jones and Knudsen (1940) developed the audiogram mirroring prescription procedure, in which every decibel (dB) of loss was compensated with an additional dB of gain [10]. The prescription was then developed by Watson and Knudsen (1940) to incorporate the individual's most comfortable level [11]. Shortly after, Lybarger (1944) developed the half-gain rule, in which every additional 2 dB of hearing loss were compensated with an additional 1 dB of gain [12]. Since then, many other prescription procedures have been developed, but very few of them have been comprehensively studied and validated. The invaluable contribution of Denis Byrne (who is known as the father of

For peer review only - http://bmjopen.bmj.com/site/about/guidelines.xhtml

prescriptive hearing aid fitting) to the development of prescription procedures has led to the indubitable universal acceptance of hearing aid prescriptions [13].

At present, several acoustic laboratories worldwide have developed validated audiogram-based prescription formulae, such as National Acoustic Laboratories Non-Linear (NAL-NL1 and NAL-NL2) [14, 15] and Desired Sensation Level (DSL5.0) [16]. These prescription formulae aimed to provide the optimal amount of amplification for each audiometric frequency [17]. Such prescriptions were revised and updated multiple times based on a combination of theoretical derivation and empirical data [18].

Rationale

Numerous direct-to-consumer hearing devices have been mass-produced and marketed to customers. Although these hearing devices vary considerably in quality, a few have comparable electroacoustics to conventional hearing aids that are programmed and fitted by audiologists [19]. Moreover, some of these hearing devices allow consumers to use their smartphone or remote controls to adjust the amplification characteristics according to their preferences [20]. Allowing users to take control of their amplification characteristics may help them to achieve a response that is better for them than one based on prescription targets that reflect what is optimal for an average person who has the same audiogram as that individual user. Alternatively, imprecision in adjustments made by the individual, or an inadequately adjustable hearing aid, may result in poorer outcomes than for hearing aids adjusted by a clinician on the basis of the individual's audiogram. In addition, individuals with hearing loss may prefer different amplification characteristics for different acoustic environments [21, 22], in ways that vary beyond the variation prescribed by existing non-linear prescription formula.

Objectives

It is unknown whether the use of individually prescribed amplification characteristics, using audiogram-based prescription formulae, and verified using real-ear probe microphone measurements, provides better outcomes than using amplification characteristics that are not prescribed on basis of the individual's audiogram (e.g., users adjust the amplification characteristics of their device based on their listening needs). Mueller (2005) systematically reviewed studies that compared audiogram-based prescriptions with each other or with the same prescription but with the user's adjustment to the overall gain [23]. Direct-to-consumer hearing devices that allow users to fine-tune the amplification characteristics without audiometric thresholds were not widely available at the time of Mueller's review. Thus, this review aims to identify whether the outcomes for adults are better when hearing aids are programmed using an audiogram-based prescription formulae.

For peer review only - http://bmjopen.bmj.com/site/about/guidelines.xhtml

METHOD AND ANALYSIS

This review protocol was pre-registered in International Prospective Register of Systematic Reviews. The method of this systematic review are reported in line with the Preferred Reporting Items for Systematic Review and Meta-Analyses (PRISMA-P) guidelines [24].

Eligibility criteria

The eligibility criteria of this review are specified and described in line with the participants, interventions, comparators, outcomes and study designs (PICOS) criteria.

Participants

Adults (≥ 18 years) with any defined degree and type of hearing loss will be included. If only qualitative descriptions of age and hearing thresholds are reported in a trial, then the study will still be included. Studies that include both adults and children will be included if the results were separately analysed and reported. In addition, studies that include participants with normal hearing and hearing loss will not be included unless they were analysed independently.

Interventions

The intervention of interest is any amplification device (i.e. conventional hearing aids or direct-to-consumer hearing devices) that has not been programmed (using either a verified or a manufacturer proprietary prescription formula) using the individual user's hearing thresholds. Amplification devices pre-programmed for generic mild or moderate hearing loss will be included, unless the experimental protocol tested the device only on people selected because they have audiograms that closely matched the generic hearing loss target(s).

Comparators

Comparators will include any amplification device (i.e. conventional hearing aids, direct-to-consumer hearing devices or simulated hearing aids) programmed for an audiogram-based prescription target (e.g. NAL-NL2) and verified using a real-ear measurement system. Implantable devices, assistive listening devices and bone conduction hearing devices will be excluded. Studies using different hearing device for the intervention and comparator will be excluded, as differences in technology, features and appearance could serve as serious confounding variables.

For peer review only - http://bmjopen.bmj.com/site/about/guidelines.xhtml

Outcomes

The primary outcome of this review is the participant's listening preference. Secondary outcomes will include hearing-specific health-related quality of life (e.g. Hearing Handicap Inventory for the Elderly [25]), self-reported listening ability (e.g. The Speech, Spatial and Qualities of Hearing Scale [26]), speech intelligibility of words and sentences in quiet and noisy situations, sound quality ratings and adverse events (e.g. discomfort, hearing aid rejection and noise-induced hearing loss). Studies reporting any of the above outcomes will be included. Studies that only quantify the deviation from the prescriptive targets will be excluded. Studies that used only predicted speech intelligibility (speech intelligibility index) as an outcome will be also be excluded.

Study designs

We will include both randomised and non-randomised controlled trials, and cross-over designs. Conference abstracts, book chapters and theses will be excluded. Case reports, reviews and clinical guidelines will also be excluded.

Information sources

A systematic search strategy was conducted to identify published, concluded but unpublished and ongoing experiments. The following databases were searched: EMBASE, MEDLINE, PubMed, PsycINFO, Web of Science, Cochrane Library, Emcare and Academic Search Premier. All databases were searched on 23 August 2020 with no search restrictions in relation to the publication's year, status and language. To ensure that this review includes the most recent evidence, the searches will be repeated at the final write-up stage.

The reference lists of the included studies will be scanned to identify other relevant studies. We will also track the citation of the included studies using Google Scholar to identify additional relevant articles.

Search strategy

A medical information specialist developed the search protocol. The search terms were developed based on free text, expert opinions and controlled terms (e.g. Medical Subject Headings). The search protocol for each of the included databases is reported in Appendix 1.

For peer review only - http://bmjopen.bmj.com/site/about/guidelines.xhtml

Study records

Data management

The search results records will be extracted to EndNote X9 Reference Management software (Clarivate Analytics, 2018) to remove duplicated records. Study details (i.e., authors, publication year, titles and abstracts) will then be transformed into a Microsoft Excel (2016) spreadsheet so that they can be easily assessed for inclusion and exclusion criteria.

Selection process

Two of the review team will independently assess the title and abstract of all retrieved studies to determine their eligibility for inclusion. The reason for excluding any article will be documented. Disagreements will be resolved by discussion or by consulting the third author. Articles that meet the inclusion criteria will be retrieved for a full inspection. The first and second authors will then screen the retrieved records against the eligibility criteria and disagreements will be resolved by discussion with the third author. The authors of all relevant identified protocols will be asked about the publication status of their clinical experiments to identify and include all studies published up until the review is completed. The study selection process along with the main reasons for exclusion will be illustrated in a PRISMA flow diagram.

Data collection process and data items

The first author will extract the data from all eligible studies. Another member of the review team will independently extract a small proportion of data to check for consistency. Any disagreements will be resolved by arbitration or by consulting the third author. An adapted version of the Cochrane Handbook data extraction form will be used to extract the data from the included studies. The main items of the data extraction are summarised in Appendix 2. When necessary, an extraction tool (e.g. WebPlotDigitizer) will be used to extract data that are only available in figures and graphs.

Risk of bias in individual studies

The methodological quality of the studies will be assessed using the Downs and Black (1998) checklist because it is easy to use and has acceptable validity and reliability. This tool provides numeric scores for each of the following domains: study quality, external validity, study bias, confounding and selection bias as well as power of the study. In this review, the score for the power domain (i.e. question number 27) will be reduced from three points to one point because there is a dearth of knowledge about the clinically important differences in hearing aid outcomes. The full score for this domain will be awarded if sample size calculation was made and a score of zero will be awarded if it was not. However, if no power analysis was performed but the sample size is

For peer review only - http://bmjopen.bmj.com/site/about/guidelines.xhtml

commensurate with the other studies for which one was performed, the study will not be penalised. Thus, the maximum possible score will be 28 and the quality of each study will be categorised as excellent (26–28), good (20–25), fair (15–19) or poor quality (<14). The assessment of the risk of bias will be carried out independently by all authors. Disagreements will be resolved by discussion or decision by majority.

Data synthesis

The data will be synthesised into a meta-analysis where possible. For between-group studies, the mean difference (when the same continuous outcomes are used across studies) or the standardised mean difference (when different continuous outcomes are used) will be calculated along with their 95% confidence interval (CI). The risk ratio and its 95% CI will be calculated for dichotomous outcomes. For crossover design studies, the effect size and its 95% CI will be calculated in accordance with Cochrane handbook recommendations, which usually require the calculation of the correlation coefficient between the evaluated interventions when the outcome is continuous variable. For the primary outcome of interest, the participants' listening preference, however, the difference in proportion between those preferring each condition and its 95% CI will be computed. A fixed-effect meta-analysis will be computed whenever the statistical heterogeneity is low; otherwise, the data will be synthesised using a random-effect meta-analysis. The generic inverse of variance approach will be used to weight each study and hence compute the effect estimate and its 95% CI. The data for each meta-analysis will be presented in a forest plot.

Asymmetrical distribution of outcomes will be assessed where either the lowest or the highest possible values of a used scale are available. The assessment involves subtracting the lowest possible value from the mean (or vice versa with the highest possible value) and dividing this by the standard deviation (SD). When the observed ratio is less than 1, there is strong evidence of a skewed distribution. A ratio between 1 and 2 also suggests a skewed distribution. Where possible, appropriate transformation methods will be used to transform and normalise the distribution of skewed data.

Data from studies that have different designs will be combined only if their effect sizes can be transferred to the same metric (e.g., standardised mean difference) and their effect sizes estimate the same treatment effect [27].

The data will be quantitatively instead of qualitatively synthesised when a meta-analysis is considered to be inappropriate.

Assessment of reporting bias

Publication bias will be assessed, when possible, using a funnel plot of the studies' standard error or precision as a function of effect estimates.

For peer review only - http://bmjopen.bmj.com/site/about/guidelines.xhtml

Assessment of heterogeneity

The variability across studies (for each outcome) will be assessed using the I^2 statistic. Percentages of 0–40%, 41–60% or 61–100% respectively indicate low, medium or high heterogeneity. In addition, a chi-squared test will be performed to assess the statistical significance of the heterogeneity.

Dealing with missing data

Missing data will be obtained by contacting the corresponding authors, where possible. Missing SDs and correlation coefficients will be estimated or imputed from the other available data using protocols explained in the Cochrane Handbook.

Subgroup analysis

Plausible sources of heterogeneity will be explored using a subgroup analysis of the participant's age, degree of hearing loss, level of experience with hearing aids and the length of their acclimatisation period with the hearing aid.

Confidence in the cumulative estimate

The Grading of Recommendations, Assessment, Development and Evaluations tool (GRADE) will be used to rate the overall evidence of each outcome. The assigned rating (i.e. high, moderate, low or very low) reflects our confidence of the pooled estimate, that is, high-quality evidence implies that the pooled estimates are probably very close to the true effect of the intervention. Conversely, very low-quality evidence implies that the pooled estimate is likely to be substantially different from the true effect and further research is needed to strengthen our confidence in the pooled effect. The initial quality ratings for randomised and non-randomised trials will be respectively high and low. Cross-over designs, where each participant acts as their own control, will also initially be assigned a high quality provided cross-over order is counterbalanced across participants. The assigned rating can be upgraded or downgraded by one or two points based on the seriousness of multiple factors. Notably, three factors may upgrade the quality of evidence: (i) large effect size, (ii) dose-response gradient (e.g., the magnitude of the participants' preference for the audiogram-based approach [comparator] increased when the deviation from the prescribed target decreased) and (iii) plausible confounding factors that likely have reduced the effect observed (e.g., participants preferred the amplification characteristics for audiogram-based approach [comparator] over the other approaches [intervention] even when more advanced features were exclusively activated with the intervention). Meanwhile, study limitations, inconsistency, indirectness, imprecision and publication bias are factors that may reduce our confidence in the pooled estimates. The overall quality rating for each outcome will be presented in a summary of findings table utilising the GRADEpro online tool (https://gradepro.org/).

For peer review only - http://bmjopen.bmj.com/site/about/guidelines.xhtml

The review team will independently grade the overall quality of each outcome. Disagreements will be resolved through discussion or consensus.

Sensitivity analysis

The impact of imputing missing data, potential confounders and studies with high risk of bias on the robustness of the findings will be assessed using a sensitivity analysis, that is, the meta-analysis will be performed twice, with and without such studies.

Patient and Public Involvement

The representatives of patients and public involvement groups were not involved in the development of this review protocol. The review findings will be disseminated to the public through social media and other public platforms.

ETHICS AND DISSEMINATION

We will only retrieve and analyse data from existing studies, hence no ethical approval is required. The review findings will be published in a peer-reviewed journal and presented in scientific conferences.

Contributor.

IA, the guarantor of this review, developed and drafted the initial version of this protocol and tested the preliminary search strategy. KJM and HD contributed to the development, critically reviewed and provided extensive feedback on the earlier versions of this manuscript. Jan Schoones (an information specialist, Systematic Review Solutions Limited) searched the databases. All authors approved this version of the manuscript.

Funding NIHR Manchester Biomedical Research Centre (funder reference: IS-BRC-1215-20007)

Competing interest None declared.

ACKNOWLEDGEMENTS

IA, KJM and HD are supported by the NIHR Manchester Biomedical research Centre. The first author is also supported by the Deanship of Scientific Research at the College of Applied Medical Sciences Research Center at King Saud University.

For peer review only - http://bmjopen.bmj.com/site/about/guidelines.xhtml

Reference

1. World Health Organization. *Deafness and hearing loss*. 2020 [cited 2019 06/10/2020]; Available from: https://www.who.int/news-room/fact-sheets/detail/deafness-and-hearing-loss.
2. World Health Organization, *Global Health Estimates 2016: Disease burden by Cause, Age, Sex, by Country and by Region, 2000-2016*. 2018: Geneva.
3. Davis, A., et al., *Acceptability, benefit and costs of early screening for hearing disability: a study of potential screening tests and models.* Health Technol Assess, 2007. **11**(42): p. 1-294.
4. Thyer, N.J., et al., *Randomised controlled feasibility trial of the Active Communication Education programme plus hearing aid provision versus hearing aid provision alone (ACE to HEAR): a study protocol.* BMJ Open, 2018. **8**(7): p. e021502.
5. Moore, B.C. and G. Popelka, *Introduction to Hearing Aid*, in *Hearing Aids*, G.R. Popelka, et al., Editors. 2016, Springer: Switzerland.
6. Kochkin, S., *MarkeTrak VIII: 25-year trends in the hearing health market.* Hearing Review, 2009. **16**(11): p. 12-31.
7. Ferguson, M.A., et al., *Hearing aids for mild to moderate hearing loss in adults.* Cochrane Database Syst Rev, 2017. **9**: p. CD012023.
8. Munro, K. and G.H. Mueller, *Clinical Verification of Hearing Aid Performance*, in *Hearing Aids*, G. Popelka, et al., Editors. 2016, Springer Switzeland.
9. Nance, G. and D. Causey, *HEARING-AID EVALUATION: AN EXAMINATION OF TWO PROCEDURES.* Bulletin of Prosthetics Research - Spring, 1968.
10. Jones, I.H. and V.O. Knudsen, *What audiometry can now mean in routine practice.* Journal of the American Medical Association, 1938. **111**(7): p. 597-605.
11. Watson, N.A. and V.O. Knudsen, *Selective Amplification in Hearing Aids.* The Journal of the Acoustical Society of America, 1940. **11**(4): p. 406-419.
12. Lybarger, S., *US patent application SN543-278.* 1944.
13. Dillon, H. *The Research of Denis Byrne at NAL: Implications for Clinicians Today*. 2001 [cited 2020 25/04]; Available from: https://www.audiologyonline.com/articles/research-denis-byrne-at-nal-1200.
14. Byrne, D., et al., *NAL-NL1 procedure for fitting nonlinear hearing aids: characteristics and comparisons with other procedures.* J Am Acad Audiol, 2001. **12**(1): p. 37-51.
15. Keidser, G., et al., *The NAL-NL2 Prescription Procedure.* Audiol Res, 2011. **1**(1): p. e24.
16. Scollie, S., et al., *The Desired Sensation Level multistage input/output algorithm.* Trends Amplif, 2005. **9**(4): p. 159-97.
17. Dillon, H., *Hearing Aids*. 2012: Thieme.
18. Keidser, G., et al., *NAL-NL2 empirical adjustments.* Trends Amplif, 2012. **16**(4): p. 211-23.
19. Almufarrij, I., et al., *Direct-to-Consumer Hearing Devices: Capabilities, Costs, and Cosmetics.* Trends Hear, 2019. **23**: p. 2331216519858301.
20. Sabin, A.T., et al., *Validation of a Self-Fitting Method for Over-the-Counter Hearing Aids.* Trends Hear, 2020. **24**: p. 2331216519900589.
21. Scollie, S., et al., *Evaluation of the NAL-NL1 and DSL v4.1 prescriptions for children: Preference in real world use.* Int J Audiol, 2010. **49 Suppl 1**: p. S49-63.
22. Walravens, E., G. Keidser, and L. Hickson, *Consistency of Hearing Aid Setting Preference in Simulated Real-World Environments: Implications for Trainable Hearing Aids.* Trends Hear, 2020. **24**: p. 2331216520933392.
23. Mueller, H.G., *Fitting hearing aids to adults using prescriptive methods: an evidence-based review of effectiveness.* J Am Acad Audiol, 2005. **16**(7): p. 448-60.
24. Shamseer, L., et al., *Preferred reporting items for systematic review and meta-analysis protocols (PRISMA-P) 2015: elaboration and explanation.* Bmj, 2015. **350**: p. g7647.

For peer review only

For peer review only - http://bmjopen.bmj.com/site/about/guidelines.xhtml

25. Ventry, I.M. and B.E. Weinstein, *The hearing handicap inventory for the elderly: a new tool.* Ear Hear, 1982. **3**(3): p. 128-34.
26. Gatehouse, S. and W. Noble, *The Speech, Spatial and Qualities of Hearing Scale (SSQ).* International journal of audiology, 2004. **43**(2): p. 85-99.
27. Morris, S.B. and R.P. DeShon, *Combining effect size estimates in meta-analysis with repeated measures and independent-groups designs.* Psychol Methods, 2002. **7**(1): p. 105-25.

For peer review only

Appendix 1: search protocols

PubMed:

(("Hearing Loss"[Mesh] OR "hearing loss"[tw] OR "hearing losses"[tw] OR "Hearing Disorders"[Mesh] OR "hearing disorder"[tw] OR "hearing disorders"[tw] OR "hearing problem"[tw] OR "hearing problems"[tw] OR "hearing impaired"[tw] OR "hearing difficulties"[tw] OR "hearing difficulty"[tw] OR "ANSD"[tw] OR "auditory neuropathy"[tw]) AND ("Hearing Aids"[Mesh] OR "hearing aid"[tw] OR "hearing aids"[tw] OR "hearing device"[tw] OR "hearing devices"[tw] OR "acoustic aid"[tw] OR "acoustic aids"[tw] OR " hearing instrument"[tw] OR " hearing instruments"[tw] OR "over-the-counter hearing"[tw] OR "over the counter hearing "[tw] OR "direct-to-consumer hearing"[tw] OR "direct to consumer hearing"[tw] OR "self-fitting hearing"[tw] OR "self fitting hearing "[tw] OR "personal sound amplification"[tw]) AND ("real ear measurement"[tw] OR "real ear measurements"[tw] OR "real ear measure"[tw] OR "real ear measures"[tw] OR "real-ear measurement"[tw] OR "real-ear measurements"[tw] OR "real-ear measure"[tw] OR "real-ear measures"[tw] OR "REM"[tw] OR "REMs"[tw] OR "PMM"[tw] OR "PMMs"[tw] OR "probe microphone"[tw] OR "probe microphones"[tw] OR "probe mic"[tw] OR "probe mics"[tw] OR "probe microphone measurement"[tw] OR "probe microphone measurements"[tw] OR "probe microphone measure"[tw] OR "probe microphone measures"[tw] OR "probemicrophone"[tw] OR "probe-microphones"[tw] OR "probe-microphone measurement"[tw] OR "probe-microphone measurements"[tw] OR "probe-microphone measure"[tw] OR "probe-microphone measures"[tw] OR "real-ear verification"[tw] OR "audiology best practices"[tw] OR "audiology best practice"[tw] OR "best practices"[tw] OR "best practice"[tw] OR "clinician-driven"[tw] OR "physician-driven"[tw] OR "doctor-driven"[tw] OR "prescriptive methods"[tw] OR "prescription methods"[tw] OR "prescriptive method*"[tw] OR "prescription method*"[tw] OR "formula"[tw] OR "formulae"[tw]))

MEDLINE (OVID)

((exp "Hearing Loss"/ OR "hearing loss".mp OR "hearing losses".mp OR exp "Hearing Disorders"/ OR "hearing disorder".mp OR "hearing disorders".mp OR "hearing problem".mp OR "hearing problems".mp OR "hearing impaired".mp OR "hearing difficulties".mp OR "hearing difficulty".mp OR "ANSD".mp OR "auditory neuropathy".mp) AND (exp "Hearing Aids"/ OR "hearing aid".mp OR "hearing aids".mp OR "hearing device".mp OR "hearing devices".mp OR "acoustic aid".mp OR "acoustic aids".mp OR " hearing instrument".mp OR " hearing instruments".mp OR "over-the-counter hearing".mp OR "over the counter hearing ".mp OR "direct-to-consumer hearing".mp OR "direct to consumer hearing".mp OR "self-fitting hearing".mp OR "self fitting hearing ".mp OR "personal sound amplification".mp) AND ("real ear measurement".mp OR "real ear measurements".mp OR "real ear measure".mp OR "real ear measures".mp OR "real-ear measurement".mp OR "real-ear measurements".mp OR "real-ear measure".mp OR "real-ear measures".mp OR "REM".mp OR "REMs".mp OR "PMM".mp OR "PMMs".mp OR "probe microphone".mp OR "probe microphones".mp OR "probe mic".mp OR "probe mics".mp OR "probe microphone measurement".mp OR "probe microphone measurements".mp OR "probe microphone measure".mp OR "probe microphone measures".mp OR "probemicrophone".mp OR "probe-microphones".mp OR "probe-microphone measurement".mp OR "probe-microphone measurements".mp OR "probe-microphone measure".mp OR "probe-microphone measures".mp OR "real-ear verification".mp OR "audiology best practices".mp OR "audiology best practice".mp OR "best practices".mp OR "best practice".mp OR "clinician-driven".mp OR "physician-driven".mp OR "doctor-driven".mp OR "prescriptive methods".mp OR "prescription methods".mp OR "prescriptive method*".mp OR "prescription method*".mp OR "formula".mp OR "formulae".mp))

For peer review only

Embase (OVID)

((exp "Hearing Impairment"/ OR "hearing loss".mp OR "hearing losses".mp OR exp "Hearing Disorder"/ OR "hearing disorder".mp OR "hearing disorders".mp OR "hearing problem".mp OR "hearing problems".mp OR "hearing impaired".mp OR "hearing difficulties".mp OR "hearing difficulty".mp OR "ANSD".mp OR "auditory neuropathy".mp) AND (exp "Hearing Aid"/ OR "hearing aid".mp OR "hearing aids".mp OR "hearing device".mp OR "hearing devices".mp OR "acoustic aid".mp OR "acoustic aids".mp OR " hearing instrument".mp OR " hearing instruments".mp OR "over-the-counter hearing".mp OR "over the counter hearing ".mp OR "direct-to-consumer hearing".mp OR "direct to consumer hearing".mp OR "self-fitting hearing".mp OR "self fitting hearing ".mp OR "personal sound amplification".mp) AND ("real ear measurement".mp OR "real ear measurements".mp OR "real ear measure".mp OR "real ear measures".mp OR "real-ear measurement".mp OR "real-ear measurements".mp OR "real-ear measure".mp OR "real-ear measures".mp OR "REM".mp OR "REMs".mp OR "PMM".mp OR "PMMs".mp OR "probe microphone".mp OR "probe microphones".mp OR "probe mic".mp OR "probe mics".mp OR "probe microphone measurement".mp OR "probe microphone measurements".mp OR "probe microphone measure".mp OR "probe microphone measures".mp OR "probemicrophone".mp OR "probe-microphones".mp OR "probe-microphone measurement".mp OR "probe-microphone measurements".mp OR "probe-microphone measure".mp OR "probe-microphone measures".mp OR "real-ear verification".mp OR "audiology best practices".mp OR "audiology best practice".mp OR "best practices".mp OR "best practice".mp OR "clinician-driven".mp OR "physician-driven".mp OR "doctor-driven".mp OR "prescriptive methods".mp OR "prescription methods".mp OR "prescriptive method*".mp OR "prescription method*".mp OR "formula".mp OR "formulae".mp))

PsycINFO

TX(("Hearing Impairment" OR "hearing loss" OR "hearing losses" OR "Hearing Disorder" OR "hearing disorder" OR "hearing disorders" OR "hearing problem" OR "hearing problems" OR "hearing impaired" OR "hearing difficulties" OR "hearing difficulty" OR "ANSD" OR "auditory neuropathy") AND ("Hearing Aid" OR "hearing aid" OR "hearing aids" OR "hearing device" OR "hearing devices" OR "acoustic aid" OR "acoustic aids" OR " hearing instrument" OR " hearing instruments" OR "over-the-counter hearing" OR "over the counter hearing " OR "direct-to-consumer hearing" OR "direct to consumer hearing" OR "self-fitting hearing" OR "self fitting hearing " OR "personal sound amplification") AND ("real ear measurement" OR "real ear measurements" OR "real ear measure" OR "real ear measures" OR "real-ear measurement" OR "real-ear measurements" OR "real-ear measure" OR "real-ear measures" OR "REM" OR "REMs" OR "PMM" OR "PMMs" OR "probe microphone" OR "probe microphones" OR "probe mic" OR "probe mics" OR "probe microphone measurement" OR "probe microphone measurements" OR "probe microphone measure" OR "probe microphone measures" OR "probemicrophone" OR "probe-microphones" OR "probe-microphone measurement" OR "probe-microphone measurements" OR "probe-microphone measure" OR "probe-microphone measures" OR "real-ear verification" OR "audiology best practices" OR "audiology best practice" OR "best practices" OR "best practice" OR "clinician-driven" OR "physician-driven" OR "doctor-driven" OR "prescriptive methods" OR "prescription methods" OR "prescriptive method*" OR "prescription method*" OR "formula" OR "formulae"))

Web of Science (all databases)

TS=(("Hearing Impairment" OR "hearing loss" OR "hearing losses" OR "Hearing Disorder" OR "hearing disorder" OR "hearing disorders" OR "hearing problem" OR "hearing problems" OR "hearing impaired" OR "hearing difficulties" OR "hearing difficulty" OR "ANSD" OR "auditory neuropathy") AND ("Hearing Aid" OR "hearing aid" OR "hearing aids" OR "hearing device" OR "hearing devices" OR "acoustic aid" OR "acoustic aids" OR " hearing instrument" OR " hearing instruments" OR "over-the-counter hearing" OR "over the counter hearing " OR "direct-to-consumer hearing" OR "direct to consumer hearing" OR "self-fitting hearing" OR "self fitting hearing " OR "personal sound amplification") AND ("real ear measurement" OR "real ear measurements" OR "real ear measure" OR "real ear measures" OR "real-ear measurement" OR "real-ear measurements" OR "real-ear measure" OR "real-ear measures" OR "REM" OR "REMs" OR "PMM" OR "PMMs" OR "probe microphone" OR "probe microphones" OR "probe mic" OR "probe mics" OR "probe microphone measurement" OR "probe microphone measurements" OR "probe microphone measure" OR "probe microphone measures" OR "probemicrophone" OR "probe-microphones" OR "probe-microphone measurement" OR "probe-microphone measurements" OR "probe-microphone measure" OR "probe-microphone measures" OR "real-ear verification" OR "audiology best practices" OR "audiology best practice" OR "best practices" OR "best practice" OR "clinician-driven" OR "physician-driven" OR "doctor-driven" OR "prescriptive methods" OR "prescription methods" OR "prescriptive method*" OR "prescription method*" OR "formula" OR "formulae"))

Cochrane Library

(("Hearing Impairment" OR "hearing loss" OR "hearing losses" OR "Hearing Disorder" OR "hearing disorder" OR "hearing disorders" OR "hearing problem" OR "hearing problems" OR "hearing impaired" OR "hearing difficulties" OR "hearing difficulty" OR "ANSD" OR "auditory neuropathy") AND ("Hearing Aid" OR "hearing aid" OR "hearing aids" OR "hearing device" OR "hearing devices" OR "acoustic aid" OR "acoustic aids" OR " hearing instrument" OR " hearing instruments" OR "over-the-counter hearing" OR "over the counter hearing " OR "direct-to-consumer hearing" OR "direct to consumer hearing" OR "self-fitting hearing" OR "self fitting hearing " OR "personal sound amplification") AND ("real ear measurement" OR "real ear measurements" OR "real ear measure" OR "real ear measures" OR "real-ear measurement" OR "real-ear measurements" OR "real-ear measure" OR "real-ear measures" OR "REM" OR "REMs" OR "PMM" OR "PMMs" OR "probe microphone" OR "probe microphones" OR "probe mic" OR "probe mics" OR "probe microphone measurement" OR "probe microphone measurements" OR "probe microphone measure" OR "probe microphone measures" OR "probemicrophone" OR "probe-microphones" OR "probe-microphone measurement" OR "probe-microphone measurements" OR "probe-microphone measure" OR "probe-microphone measures" OR "real-ear verification" OR "audiology best practices" OR "audiology best practice" OR "best practices" OR "best practice" OR "clinician-driven" OR "physician-driven" OR "doctor-driven" OR "prescriptive methods" OR "prescription methods" OR "prescriptive method*" OR "prescription method*" OR "formula" OR "formulae")):ti,ab,kw

Emcare

((exp "Hearing Impairment"/ OR "hearing loss".mp OR "hearing losses".mp OR exp "Hearing Disorder"/ OR "hearing disorder".mp OR "hearing disorders".mp OR "hearing problem".mp OR "hearing problems".mp OR "hearing impaired".mp OR "hearing difficulties".mp OR "hearing difficulty".mp OR "ANSD".mp OR "auditory neuropathy".mp) AND (exp "Hearing Aid"/ OR "hearing aid".mp OR "hearing aids".mp OR "hearing device".mp OR "hearing devices".mp OR

For peer review only

"acoustic aid".mp OR "acoustic aids".mp OR " hearing instrument".mp OR " hearing instruments".mp OR "over-the-counter hearing".mp OR "over the counter hearing ".mp OR "direct-to-consumer hearing".mp OR "direct to consumer hearing".mp OR "self-fitting hearing".mp OR "self fitting hearing ".mp OR "personal sound amplification".mp) AND ("real ear measurement".mp OR "real ear measurements".mp OR "real ear measure".mp OR "real ear measures".mp OR "real-ear measurement".mp OR "real-ear measurements".mp OR "real-ear measure".mp OR "real-ear measures".mp OR "REM".mp OR "REMs".mp OR "PMM".mp OR "PMMs".mp OR "probe microphone".mp OR "probe microphones".mp OR "probe mic".mp OR "probe mics".mp OR "probe microphone measurement".mp OR "probe microphone measurements".mp OR "probe microphone measure".mp OR "probe microphone measures".mp OR "probemicrophone".mp OR "probe-microphones".mp OR "probe-microphone measurement".mp OR "probe-microphone measurements".mp OR "probe-microphone measure".mp OR "probe-microphone measures".mp OR "real-ear verification".mp OR "audiology best practices".mp OR "audiology best practice".mp OR "best practices".mp OR "best practice".mp OR "clinician-driven".mp OR "physician-driven".mp OR "doctor-driven".mp OR "prescriptive methods".mp OR "prescription methods".mp OR "prescriptive method*".mp OR "prescription method*".mp OR "formula".mp OR "formulae".mp))

Academic Search Premier

TI(("Hearing Impairment" OR "hearing loss" OR "hearing losses" OR "Hearing Disorder" OR "hearing disorder" OR "hearing disorders" OR "hearing problem" OR "hearing problems" OR "hearing impaired" OR "hearing difficulties" OR "hearing difficulty" OR "ANSD" OR "auditory neuropathy") AND ("Hearing Aid" OR "hearing aid" OR "hearing aids" OR "hearing device" OR "hearing devices" OR "acoustic aid" OR "acoustic aids" OR " hearing instrument" OR " hearing instruments" OR "over-the-counter hearing" OR "over the counter hearing " OR "direct-to-consumer hearing" OR "direct to consumer hearing" OR "self-fitting hearing" OR "self fitting hearing " OR "personal sound amplification") AND ("real ear measurement" OR "real ear measurements" OR "real ear measure" OR "real ear measures" OR "real-ear measurement" OR "real-ear measurements" OR "real-ear measure" OR "real-ear measures" OR "REM" OR "REMs" OR "PMM" OR "PMMs" OR "probe microphone" OR "probe microphones" OR "probe mic" OR "probe mics" OR "probe microphone measurement" OR "probe microphone measurements" OR "probe microphone measure" OR "probe microphone measures" OR "probemicrophone" OR "probe-microphones" OR "probe-microphone measurement" OR "probe-microphone measurements" OR "probe-microphone measure" OR "probe-microphone measures" OR "real-ear verification" OR "audiology best practices" OR "audiology best practice" OR "best practices" OR "best practice" OR "clinician-driven" OR "physician-driven" OR "doctor-driven" OR "prescriptive methods" OR "prescription methods" OR "prescriptive method*" OR "prescription method*" OR "formula" OR "formulae")) OR SU(("Hearing Impairment" OR "hearing loss" OR "hearing losses" OR "Hearing Disorder" OR "hearing disorder" OR "hearing disorders" OR "hearing problem" OR "hearing problems" OR "hearing impaired" OR "hearing difficulties" OR "hearing difficulty" OR "ANSD" OR "auditory neuropathy") AND ("Hearing Aid" OR "hearing aid" OR "hearing aids" OR "hearing device" OR "hearing devices" OR "acoustic aid" OR "acoustic aids" OR " hearing instrument" OR " hearing instruments" OR "over-the-counter hearing" OR "over the counter hearing " OR "direct-to-consumer hearing" OR "direct to consumer hearing" OR "self-fitting hearing" OR "self fitting hearing " OR "personal sound amplification") AND ("real ear measurement" OR "real ear measurements" OR "real ear measure" OR "real ear measures" OR "real-ear measurement" OR "real-ear measurements" OR "real-ear measure" OR "real-ear measures" OR "REM" OR "REMs" OR "PMM" OR "PMMs" OR "probe microphone" OR "probe microphones" OR "probe mic" OR "probe mics" OR "probe microphone measurement" OR "probe microphone measurements" OR "probe microphone measure" OR "probe microphone

For peer review only

measures" OR "probemicrophone" OR "probe-microphones" OR "probe-microphone measurement" OR "probe-microphone measurements" OR "probe-microphone measure" OR "probe-microphone measures" OR "real-ear verification" OR "audiology best practices" OR "audiology best practice" OR "best practices" OR "best practice" OR "clinician-driven" OR "physician-driven" OR "doctor-driven" OR "prescriptive methods" OR "prescription methods" OR "prescriptive method*" OR "prescription method*" OR "formula" OR "formulae")) OR KW(("Hearing Impairment" OR "hearing loss" OR "hearing losses" OR "Hearing Disorder" OR "hearing disorder" OR "hearing disorders" OR "hearing problem" OR "hearing problems" OR "hearing impaired" OR "hearing difficulties" OR "hearing difficulty" OR "ANSD" OR "auditory neuropathy") AND ("Hearing Aid" OR "hearing aid" OR "hearing aids" OR "hearing device" OR "hearing devices" OR "acoustic aid" OR "acoustic aids" OR " hearing instrument" OR " hearing instruments" OR "over-the-counter hearing" OR "over the counter hearing " OR "direct-to-consumer hearing" OR "direct to consumer hearing" OR "self-fitting hearing" OR "self fitting hearing " OR "personal sound amplification") AND ("real ear measurement" OR "real ear measurements" OR "real ear measure" OR "real ear measures" OR "real-ear measurement" OR "real-ear measurements" OR "real-ear measure" OR "real-ear measures" OR "REM" OR "REMs" OR "PMM" OR "PMMs" OR "probe microphone" OR "probe microphones" OR "probe mic" OR "probe mics" OR "probe microphone measurement" OR "probe microphone measurements" OR "probe microphone measure" OR "probe microphone measures" OR "probemicrophone" OR "probe-microphones" OR "probe-microphone measurement" OR "probe-microphone measurements" OR "probe-microphone measure" OR "probe-microphone measures" OR "real-ear verification" OR "audiology best practices" OR "audiology best practice" OR "best practices" OR "best practice" OR "clinician-driven" OR "physician-driven" OR "doctor-driven" OR "prescriptive methods" OR "prescription methods" OR "prescriptive method*" OR "prescription method*" OR "formula" OR "formulae")) OR AB(("Hearing Impairment" OR "hearing loss" OR "hearing losses" OR "Hearing Disorder" OR "hearing disorder" OR "hearing disorders" OR "hearing problem" OR "hearing problems" OR "hearing impaired" OR "hearing difficulties" OR "hearing difficulty" OR "ANSD" OR "auditory neuropathy") AND ("Hearing Aid" OR "hearing aid" OR "hearing aids" OR "hearing device" OR "hearing devices" OR "acoustic aid" OR "acoustic aids" OR " hearing instrument" OR " hearing instruments" OR "over-the-counter hearing" OR "over the counter hearing " OR "direct-to-consumer hearing" OR "direct to consumer hearing" OR "self-fitting hearing" OR "self fitting hearing " OR "personal sound amplification") AND ("real ear measurement" OR "real ear measurements" OR "real ear measure" OR "real ear measures" OR "real-ear measurement" OR "real-ear measurements" OR "real-ear measure" OR "real-ear measures" OR "REM" OR "REMs" OR "PMM" OR "PMMs" OR "probe microphone" OR "probe microphones" OR "probe mic" OR "probe mics" OR "probe microphone measurement" OR "probe microphone measurements" OR "probe microphone measure" OR "probe microphone measures" OR "probemicrophone" OR "probe-microphones" OR "probe-microphone measurement" OR "probe-microphone measurements" OR "probe-microphone measure" OR "probe-microphone measures" OR "real-ear verification" OR "audiology best practices" OR "audiology best practice" OR "best practices" OR "best practice" OR "clinician-driven" OR "physician-driven" OR "doctor-driven" OR "prescriptive methods" OR "prescription methods" OR "prescriptive method*" OR "prescription method*" OR "formula" OR "formulae"))

For peer review only

Appendix 2: Data items

General information	Method	Participants	Intervention	Outcome	Funding source	Declaration of interest	Additional comments
Title<br>Authors<br>Publication year	Study design<br>Conditions and the full study durations<br>Sequence generation<br>Sequence concealment<br>Blinding<br>Power analysis	Sample size<br>Country / Setting<br>Age<br>Sex<br>Inclusion and exclusion criteria	Intervention group<br>Comparator group	Primary outcome<br>Secondary outcome(s)<br>Adverse events			

For peer review only

Preferred reporting items for systematic review and meta-analysis protocols (PRISMA-P) 2015

PRISMA-P 2015 Checklist

Section/topic	Item No	Checklist item	Information reported Yes	Information reported No	Page number
**ADMINISTRATIVE INFORMATION**					
**Title**					
Identification	1a	Identify the report as a protocol of a systematic review	X		1
Update	1b	If the protocol is for an update of a previous systematic review, identify as such		X	Not an update
**Registration**	2	If registered, provide the name of the registry (e.g., PROSPERO) and registration number in the Abstract	X		2
**Authors**					
Contact	3a	Provide name, institutional affiliation, and e-mail address of all protocol authors; provide physical mailing address of corresponding author	X		1
Contributions	3b	Describe contributions of protocol authors and identify the guarantor of the review	X		10
**Amendments**	4	If the protocol represents an amendment of a previously completed or published protocol, identify as such and list changes; otherwise, state plan for documenting important protocol amendments		X	Not an amendment
**Support**					
Sources	5a	Indicate sources of financial or other support for the review	X		10
Sponsor	5b	Provide name for the review funder and/or sponsor	X		10
Role of sponsor/funder	5c	Describe roles of funder(s), sponsor(s), and/or institution(s), if any, in developing the protocol	X		10
**INTRODUCTION**					
**Rationale**	6	Describe the rationale for the review in the context of what is already known	X		4
**Objectives**	7	Provide an explicit statement of the question(s) the review will address with reference to participants, interventions, comparators, and outcomes (PICO)	X		4-5
**METHODS**					

For peer review only

**Eligibility criteria**	8	Specify the study characteristics (e.g., PICO, study design, setting, time frame) and report characteristics (e.g., years considered, language, publication status) to be used as criteria for eligibility for the review	☒	☐	5-6
**Information sources**	9	Describe all intended information sources (e.g., electronic databases, contact with study authors, trial registers, or other grey literature sources) with planned dates of coverage	☒	☐	6
**Search strategy**	10	Present draft of search strategy to be used for at least one electronic database, including planned limits, such that it could be repeated	☒	☐	Appendix 1
STUDY RECORDS					
Data management	11a	Describe the mechanism(s) that will be used to manage records and data throughout the review	☒	☐	6-7
Selection process	11b	State the process that will be used for selecting studies (e.g., two independent reviewers) through each phase of the review (i.e., screening, eligibility, and inclusion in meta-analysis)	☒	☐	7
Data collection process	11c	Describe planned method of extracting data from reports (e.g., piloting forms, done independently, in duplicate), any processes for obtaining and confirming data from investigators	☒	☐	7
**Data items**	12	List and define all variables for which data will be sought (e.g., PICO items, funding sources), any pre-planned data assumptions and simplifications	☒	☐	7 and Appendix 2
**Outcomes and prioritization**	13	List and define all outcomes for which data will be sought, including prioritization of main and additional outcomes, with rationale	☒	☐	6
**Risk of bias in individual studies**	14	Describe anticipated methods for assessing risk of bias of individual studies, including whether this will be done at the outcome or study level, or both; state how this information will be used in data synthesis	☒	☐	7
DATA					
**Synthesis**	15a	Describe criteria under which study data will be quantitatively synthesized	☒	☐	8
	15b	If data are appropriate for quantitative synthesis, describe planned summary measures, methods of handling data, and methods of combining data from studies, including any planned exploration of consistency (e.g., I^2, Kendall's tau)	☒	☐	8
	15c	Describe any proposed additional analyses (e.g., sensitivity or subgroup analyses, meta-regression)	☒	☐	9
	15d	If quantitative synthesis is not appropriate, describe the type of summary planned	☒	☐	8
**Meta-bias(es)**	16	Specify any planned assessment of meta-bias(es) (e.g., publication bias across studies, selective reporting within studies)	☒	☐	8-9
**Confidence in cumulative evidence**	17	Describe how the strength of the body of evidence will be assessed (e.g., GRADE)	☒	☐	9

For peer review only

BMJ Open

Is the outcome of fitting hearing aids to adults affected by whether an audiogram-based prescription formula is individually applied? A systematic review protocol.

Journal:	*BMJ Open*
Manuscript ID	bmjopen-2020-045899.R2
Article Type:	Protocol
Date Submitted by the Author:	15-Apr-2021
Complete List of Authors:	Almufarrij, Ibrahim; The University of Manchester, Manchester Centre for Audiology and Deafness, School of Health Sciences; King Saud University, Department of Rehabilitation Sciences<br>Dillon, Harvey; The University of Manchester, Manchester Centre for Audiology and Deafness, School of Health Sciences; Macquarie University, Department of Linguistics<br>Munro, Kevin; The University of Manchester, Manchester Centre for Audiology and Deafness, School of Health Sciences; Manchester University NHS Foundation Trust, Manchester University Hospitals NHS Foundation Trust, Manchester Academic Health Science Centre,
**Primary Subject Heading**:	Ear, nose and throat/otolaryngology
Secondary Subject Heading:	Communication, Ear, nose and throat/otolaryngology, Evidence based practice
Keywords:	Audiology < OTOLARYNGOLOGY, Neurotology < OTOLARYNGOLOGY, Adult otolaryngology < OTOLARYNGOLOGY

SCHOLARONE™
Manuscripts

I, the Submitting Author has the right to grant and does grant on behalf of all authors of the Work (as defined in the below author licence), an exclusive licence and/or a non-exclusive licence for contributions from authors who are: i) UK Crown employees; ii) where BMJ has agreed a CC-BY licence shall apply, and/or iii) in accordance with the terms applicable for US Federal Government officers or employees acting as part of their official duties; on a worldwide, perpetual, irrevocable, royalty-free basis to BMJ Publishing Group Ltd ("BMJ") its licensees and where the relevant Journal is co-owned by BMJ to the co-owners of the Journal, to publish the Work in this journal and any other BMJ products and to exploit all rights, as set out in our licence.

The Submitting Author accepts and understands that any supply made under these terms is made by BMJ to the Submitting Author unless you are acting as an employee on behalf of your employer or a postgraduate student of an affiliated institution which is paying any applicable article publishing charge ("APC") for Open Access articles. Where the Submitting Author wishes to make the Work available on an Open Access basis (and intends to pay the relevant APC), the terms of reuse of such Open Access shall be governed by a Creative Commons licence – details of these licences and which Creative Commons licence will apply to this Work are set out in our licence referred to above.

Other than as permitted in any relevant BMJ Author's Self Archiving Policies, I confirm this Work has not been accepted for publication elsewhere, is not being considered for publication elsewhere and does not duplicate material already published. I confirm all authors consent to publication of this Work and authorise the granting of this licence.

For peer review only

Is the outcome of fitting hearing aids to adults affected by whether an audiogram-based prescription formula is individually applied? A systematic review protocol.

Authors: Ibrahim Almufarrij[1, 2], Harvey Dillon[1,4] and Kevin J Munro[1, 3]

Author affiliations

[1] Manchester Centre for Audiology and Deafness, School of Health Sciences, University of Manchester and the Manchester Academic Health Science Centre, UK

[2] Department of Rehabilitation Sciences, College of Applied Medical Sciences, King Saud University, Riyadh, Kingdom of Saudi Arabia

[3] Manchester University Hospitals NHS Foundation Trust, Manchester Academic Health Science Centre, UK

[4] Department of Linguistics, Macquarie University, Sydney, Australia

Corresponding author: Ibrahim Almufarrij, Manchester Centre for Audiology and Deafness, University of Manchester, Manchester, M13 9PL, United Kingdom. E-mail: Email: ialmufarrij@ksu.edu.sa

For peer review only - http://bmjopen.bmj.com/site/about/guidelines.xhtml

ABSTRACT

Introduction: Hearing aids are typically programmed using the individual's audiometric thresholds. Developments in technology have resulted in a new category of direct-to-consumer devices, which are not programmed using the individual's audiometric thresholds. This review aims to identify whether programming hearing aids using the individual's audiogram-based prescription results in better outcomes for adults with hearing loss.

Methods and analysis: The methods of this review are reported in line with Preferred Reporting Items for Systematic Reviews and Meta-Analyses Protocols guidelines. On 23 August 2020, eight different databases were systematically searched without any restrictions: EMBASE, MEDLINE, PubMed, PsycINFO, Web of Science, Cochrane Library, Emcare and Academic Search Premier. To ensure that this review includes the most recent evidence, the searches will be repeated at the final write-up stage. The population of interest of this review will be adults with any degree or type of hearing loss. The studies should compare hearing aids programmed using an audiogram-based prescription (and verified in the real ear) to those not programmed on the basis of the individual's audiogram. The primary outcome of interest is consumers' listening preferences. Hearing-specific health-related quality of life, self-reported listening ability, speech intelligibility of words and sentences in quiet and noisy situations, sound quality ratings and adverse events are the secondary outcomes of interest. Both randomised and non-randomised controlled trials will be included. The quality of each individual study and the overall evidence will be assessed using Down and Black's checklist and the Grading of Recommendations, Assessment, Development and Evaluations tool, respectively.

Ethics and dissemination: We will only retrieve and analyse data from published studies, so no ethical approval is required. The review findings will be published in a peer-reviewed journal and presented at scientific conferences.

PROSPERO registration number: CRD42020197232

For peer review only - http://bmjopen.bmj.com/site/about/guidelines.xhtml

Strengths and limitations of the study:

- The primary outcome is listening preference, measured in a within-group (cross-over) experiment, but both within- and between-group design studies will be included as the latter design can contribute to secondary outcomes.
- Amplification devices pre-programmed for generic mild or moderate hearing loss will be included amongst the non-individually programmed devices.
- We will only include studies that compare programming approaches using the same hearing device.
- Magazine articles, conference abstracts, clinical guidelines and theses will be excluded.
- The results will help decision makers and hearing health professionals to provide better patient-centred care.

INTRODUCTION

Hearing loss is a debilitating health condition that affects more than 450 million individuals worldwide [1]. It is also the most common cause for years lived with disability [2]. Hearing loss can directly affect people's ability to communicate, which can consequently lead to depression and poor social interaction [3]. It is also associated with a reduced quality of life and an increased risk of dementia [4]. Hearing loss can be partially and successfully mitigated through the use of complex electroacoustic amplification devices known as hearing aids [5].

Hearing aids are the primary and the most common clinical intervention for hearing loss because they are very effective in improving hearing-related quality of life [6, 7]. The ultimate goals for hearing aids are to restore the audibility of soft level sounds, maximise the intelligibility of conversational-level sounds and ensure that loud sounds are within comfortable levels [8].

In the early 1940s and 1950s, the most controversial aspects of clinical audiology were hearing aid selection and fitting, as experts could not agree upon the most appropriate approaches [9]. Since this period, prescriptive approaches have evolved to provide recommended gains for each audiometric frequency and input level. The concept of hearing aid prescriptions goes back to the early 1940s when Jones and Knudsen (1940) developed the audiogram mirroring prescription procedure, in which every decibel (dB) of loss was compensated with an additional dB of gain [10]. The prescription was then developed by Watson and Knudsen (1940) to incorporate the individual's most comfortable level [11]. Shortly after, Lybarger (1944) developed the half-gain rule, in which every

For peer review only - http://bmjopen.bmj.com/site/about/guidelines.xhtml

additional 2 dB of hearing loss were compensated with an additional 1 dB of gain [12]. Since then, many other prescription procedures have been developed, but very few of them have been comprehensively studied and validated. The invaluable contribution of Denis Byrne (who is known as the father of prescriptive hearing aid fitting) to the development of prescription procedures has led to the indubitable universal acceptance of hearing aid prescriptions [13].

At present, several acoustic laboratories worldwide have developed validated audiogram-based prescription formulae, such as National Acoustic Laboratories Non-Linear (NAL-NL1 and NAL-NL2) [14, 15] and Desired Sensation Level (DSL5.0) [16]. These prescription formulae aimed to provide the optimal amount of amplification for each audiometric frequency [17]. Such prescriptions were revised and updated multiple times based on a combination of theoretical derivation and empirical data [18].

Rationale

Numerous direct-to-consumer hearing devices have been mass-produced and marketed to customers. Although these hearing devices vary considerably in quality, a few have comparable electroacoustics to conventional hearing aids that are programmed and fitted by audiologists [19]. Moreover, some of these hearing devices allow consumers to use their smartphone or remote controls to adjust the amplification characteristics according to their preferences [20]. Allowing users to take control of their amplification characteristics may help them to achieve a response that is better for them than one based on prescription targets that reflect what is optimal for an average person who has the same audiogram as that individual user. Alternatively, imprecision in adjustments made by the individual, or an inadequately adjustable hearing aid, may result in poorer outcomes than for hearing aids adjusted by a clinician on the basis of the individual's audiogram. In addition, individuals with hearing loss may prefer different amplification characteristics for different acoustic environments [21, 22], in ways that vary beyond the variation prescribed by existing non-linear prescription formula.

Objectives

It is unknown whether the use of individually prescribed amplification characteristics, using audiogram-based prescription formulae, and verified using real-ear probe microphone measurements, provides better outcomes than using amplification characteristics that are not prescribed on basis of the individual's audiogram (e.g., users adjust the amplification characteristics of their device based

For peer review only - http://bmjopen.bmj.com/site/about/guidelines.xhtml
For peer review only

on their listening needs). Mueller (2005) systematically reviewed studies that compared audiogram-based prescriptions with each other or with the same prescription but with the user's adjustment to the overall gain [23]. Direct-to-consumer hearing devices that allow users to fine-tune the amplification characteristics without audiometric thresholds were not widely available at the time of Mueller's review. Thus, this review aims to identify whether the outcomes for adults are better when hearing aids are programmed using an audiogram-based prescription formulae.

METHOD AND ANALYSIS

This review protocol was pre-registered in International Prospective Register of Systematic Reviews. The method of this systematic review are reported in line with the Preferred Reporting Items for Systematic Review and Meta-Analyses (PRISMA-P) guidelines [24].

Eligibility criteria

The eligibility criteria of this review are specified and described in line with the participants, interventions, comparators, outcomes and study designs (PICOS) criteria.

Participants

Adults (≥ 18 years) with any defined degree and type of hearing loss will be included. If only qualitative descriptions of age and hearing thresholds are reported in a trial, then the study will still be included. Studies that include both adults and children will be included if the results were separately analysed and reported. In addition, studies that include participants with normal hearing and hearing loss will not be included unless they were analysed independently.

Interventions

The intervention of interest is any amplification device (i.e. conventional hearing aids, direct-to-consumer hearing devices or simulated hearing aids) that has not been programmed (using either a verified or a manufacturer proprietary prescription formula) using the individual user's hearing thresholds. Amplification devices pre-programmed for generic mild or moderate hearing loss will be included, unless the experimental protocol tested the device only on people selected because they have audiograms that closely matched the generic hearing loss target(s).

The initial plan was to exclude studies that used simulated hearing aids because they are less likely to reflect real-life benefit. However, to maximise the number of included studies, we broadened the inclusion criteria to include simulated hearing aids.

For peer review only - http://bmjopen.bmj.com/site/about/guidelines.xhtml

Comparators

Comparators will include any amplification device (i.e. conventional hearing aids, direct-to-consumer hearing devices or simulated hearing aids) programmed for an audiogram-based prescription target (e.g. NAL-NL2) and verified using a real-ear measurement system. Implantable devices, assistive listening devices and bone conduction hearing devices will be excluded. Studies using different hearing device for the intervention and comparator will be excluded, as differences in technology, features and appearance could serve as serious confounding variables.

Outcomes

The primary outcome of this review is the participant's listening preference. Secondary outcomes will include hearing-specific health-related quality of life (e.g. Hearing Handicap Inventory for the Elderly [25]), self-reported listening ability (e.g. The Speech, Spatial and Qualities of Hearing Scale [26]), speech intelligibility of words and sentences in quiet and noisy situations, sound quality ratings and adverse events (e.g. discomfort, hearing aid rejection and noise-induced hearing loss). Studies reporting any of the above outcomes will be included. Studies that only quantify the deviation from the prescriptive targets will be excluded. Studies that used only predicted speech intelligibility (speech intelligibility index) as an outcome will be also be excluded.

Study designs

We will include both randomised and non-randomised controlled trials, and cross-over designs. Conference abstracts, book chapters and theses will be excluded. Case reports, reviews and clinical guidelines will also be excluded.

Information sources

A systematic search strategy was conducted to identify published, concluded but unpublished and ongoing experiments. The following databases were searched: EMBASE, MEDLINE, PubMed, PsycINFO, Web of Science, Cochrane Library, Emcare and Academic Search Premier. All databases were searched on 23 August 2020 with no search restrictions in relation to the publication's year, status and language. To ensure that this review includes the most recent evidence, the searches will be repeated at the final write-up stage. The initial plan was to include grey literature in the information sources, but they were excluded because a preliminary search produced no relevant records, and there is no agreed method of systematically searching such literature.

For peer review only - http://bmjopen.bmj.com/site/about/guidelines.xhtml

The reference lists of the included studies will be scanned to identify other relevant studies. We will also track the citation of the included studies using Google Scholar to identify additional relevant articles.

Search strategy

A medical information specialist developed the search protocol. The search terms were developed based on free text, expert opinions and controlled terms (e.g. Medical Subject Headings). The search protocol for each of the included databases is reported in Appendix 1.

Study records

Data management

The search results records will be extracted to EndNote X9 Reference Management software (Clarivate Analytics, 2018) to remove duplicated records. Study details (i.e., authors, publication year, titles and abstracts) will then be transformed into a Microsoft Excel (2016) spreadsheet so that they can be easily assessed for inclusion and exclusion criteria.

Selection process

Two of the review team will independently assess the title and abstract of all retrieved studies to determine their eligibility for inclusion. The reason for excluding any article will be documented. Disagreements will be resolved by discussion or by consulting the third author. Articles that meet the inclusion criteria will be retrieved for a full inspection. The first and second authors will then screen the retrieved records against the eligibility criteria and disagreements will be resolved by discussion with the third author. The authors of all relevant identified protocols will be asked about the publication status of their clinical experiments to identify and include all studies published up until the review is completed. The study selection process along with the main reasons for exclusion will be illustrated in a PRISMA flow diagram.

Data collection process and data items

The first author will extract the data from all eligible studies. Another member of the review team will independently extract a small proportion of data to check for consistency. Any disagreements will be resolved by arbitration or by consulting the third author. An adapted version of the Cochrane

For peer review only - http://bmjopen.bmj.com/site/about/guidelines.xhtml

Handbook data extraction form will be used to extract the data from the included studies. The main items of the data extraction are summarised in Appendix 2. When necessary, an extraction tool (e.g. WebPlotDigitizer) will be used to extract data that are only available in figures and graphs.

Risk of bias in individual studies

The methodological quality of the studies will be assessed using the Downs and Black (1998) checklist because it is easy to use and has acceptable validity and reliability. This tool provides numeric scores for each of the following domains: study quality, external validity, study bias, confounding and selection bias as well as power of the study. In this review, the score for the power domain (i.e. question number 27) will be reduced from three points to one point because there is a dearth of knowledge about the clinically important differences in hearing aid outcomes. The full score for this domain will be awarded if sample size calculation was made and a score of zero will be awarded if it was not. However, if no power analysis was performed but the sample size is commensurate with the other studies for which one was performed, the study will not be penalised. Thus, the maximum possible score will be 28 and the quality of each study will be categorised as excellent (26–28), good (20–25), fair (15–19) or poor quality (<14). The assessment of the risk of bias will be carried out independently by all authors. Disagreements will be resolved by discussion or decision by majority.

Data synthesis

The data will be synthesised into a meta-analysis where possible. For between-group studies, the mean difference (when the same continuous outcomes are used across studies) or the standardised mean difference (when different continuous outcomes are used) will be calculated along with their 95% confidence interval (CI). The risk ratio and its 95% CI will be calculated for dichotomous outcomes. For crossover design studies, the effect size and its 95% CI will be calculated in accordance with Cochrane handbook recommendations, which usually require the calculation of the correlation coefficient between the evaluated interventions when the outcome is continuous variable. For the primary outcome of interest, the participants' listening preference, however, the difference in proportion between those preferring each condition and its 95% CI will be computed. A fixed-effect meta-analysis will be computed whenever the statistical heterogeneity is low; otherwise, the data will be synthesised using a random-effect meta-analysis. The generic inverse of variance approach will be

For peer review only - http://bmjopen.bmj.com/site/about/guidelines.xhtml

used to weight each study and hence compute the effect estimate and its 95% CI. The data for each meta-analysis will be presented in a forest plot.

Asymmetrical distribution of outcomes will be assessed where either the lowest or the highest possible values of a used scale are available. The assessment involves subtracting the lowest possible value from the mean (or vice versa with the highest possible value) and dividing this by the standard deviation (SD). When the observed ratio is less than 1, there is strong evidence of a skewed distribution. A ratio between 1 and 2 also suggests a skewed distribution. Where possible, appropriate transformation methods will be used to transform and normalise the distribution of skewed data.

Data from studies that have different designs will be combined only if their effect sizes can be transferred to the same metric (e.g., standardised mean difference) and their effect sizes estimate the same treatment effect [27].

The data will be quantitatively instead of qualitatively synthesised when a meta-analysis is considered to be inappropriate.

Assessment of reporting bias

Publication bias will be assessed, when possible, using a funnel plot of the studies' standard error or precision as a function of effect estimates.

Assessment of heterogeneity

The variability across studies (for each outcome) will be assessed using the I^2 statistic. Percentages of 0–40%, 41–60% or 61–100% respectively indicate low, medium or high heterogeneity. In addition, a chi-squared test will be performed to assess the statistical significance of the heterogeneity.

Dealing with missing data

Missing data will be obtained by contacting the corresponding authors, where possible. Missing SDs and correlation coefficients will be estimated or imputed from the other available data using protocols explained in the Cochrane Handbook.

For peer review only - http://bmjopen.bmj.com/site/about/guidelines.xhtml

For peer review only

Subgroup analysis

Plausible sources of heterogeneity will be explored using a subgroup analysis of the participant's age, degree of hearing loss, level of experience with hearing aids and the length of their acclimatisation period with the hearing aid.

Confidence in the cumulative estimate

The Grading of Recommendations, Assessment, Development and Evaluations tool (GRADE) will be used to rate the overall evidence of each outcome. The assigned rating (i.e. high, moderate, low or very low) reflects our confidence of the pooled estimate, that is, high-quality evidence implies that the pooled estimates are probably very close to the true effect of the intervention. Conversely, very low-quality evidence implies that the pooled estimate is likely to be substantially different from the true effect and further research is needed to strengthen our confidence in the pooled effect. The initial quality ratings for randomised and non-randomised trials will be respectively high and low. Cross-over designs, where each participant acts as their own control, will also initially be assigned a high quality provided cross-over order is counterbalanced across participants. The assigned rating can be upgraded or downgraded by one or two points based on the seriousness of multiple factors. Notably, three factors may upgrade the quality of evidence: (i) large effect size, (ii) dose-response gradient (e.g., the magnitude of the participants' preference for the audiogram-based approach [comparator] increased when the deviation from the prescribed target decreased) and (iii) plausible confounding factors that likely have reduced the effect observed (e.g., participants preferred the amplification characteristics for audiogram-based approach [comparator] over the other approaches [intervention] even when more advanced features were exclusively activated with the intervention). Meanwhile, study limitations, inconsistency, indirectness, imprecision and publication bias are factors that may reduce our confidence in the pooled estimates. The overall quality rating for each outcome will be presented in a summary of findings table utilising the GRADEpro online tool (https://gradepro.org/). The review team will independently grade the overall quality of each outcome. Disagreements will be resolved through discussion or consensus.

For peer review only - http://bmjopen.bmj.com/site/about/guidelines.xhtml

Sensitivity analysis

The impact of imputing missing data, potential confounders and studies with high risk of bias on the robustness of the findings will be assessed using a sensitivity analysis, that is, the meta-analysis will be performed twice, with and without such studies.

Patient and Public Involvement

The representatives of patients and public involvement groups were not involved in the development of this review protocol. The review findings will be disseminated to the public through social media and other public platforms.

ETHICS AND DISSEMINATION

We will only retrieve and analyse data from existing studies, hence no ethical approval is required. The review findings will be published in a peer-reviewed journal and presented in scientific conferences.

Contributor.

IA, the guarantor of this review, developed and drafted the initial version of this protocol and tested the preliminary search strategy. KJM and HD contributed to the development, critically reviewed and provided extensive feedback on the earlier versions of this manuscript. Jan Schoones (an information specialist, Systematic Review Solutions Limited) searched the databases. All authors approved this version of the manuscript.

Funding NIHR Manchester Biomedical Research Centre (funder reference: IS-BRC-1215-20007)

Competing interest None declared.

ACKNOWLEDGEMENTS

IA, KJM and HD are supported by the NIHR Manchester Biomedical research Centre. The first author is also supported by the Deanship of Scientific Research at the College of Applied Medical Sciences Research Center at King Saud University.

For peer review only - http://bmjopen.bmj.com/site/about/guidelines.xhtml

For peer review only

Reference

1. World Health Organization. *Deafness and hearing loss*. 2020 [cited 2019 06/10/2020]; Available from: https://www.who.int/news-room/fact-sheets/detail/deafness-and-hearing-loss.
2. World Health Organization, *Global Health Estimates 2016: Disease burden by Cause, Age, Sex, by Country and by Region, 2000-2016*. 2018: Geneva.
3. Davis, A., et al., *Acceptability, benefit and costs of early screening for hearing disability: a study of potential screening tests and models.* Health Technol Assess, 2007. **11**(42): p. 1-294.
4. Thyer, N.J., et al., *Randomised controlled feasibility trial of the Active Communication Education programme plus hearing aid provision versus hearing aid provision alone (ACE to HEAR): a study protocol.* BMJ Open, 2018. **8**(7): p. e021502.
5. Moore, B.C. and G. Popelka, *Introduction to Hearing Aid*, in *Hearing Aids*, G.R. Popelka, et al., Editors. 2016, Springer: Switzerland.
6. Kochkin, S., *MarkeTrak VIII: 25-year trends in the hearing health market.* Hearing Review, 2009. **16**(11): p. 12-31.
7. Ferguson, M.A., et al., *Hearing aids for mild to moderate hearing loss in adults.* Cochrane Database Syst Rev, 2017. **9**: p. CD012023.
8. Munro, K. and G.H. Mueller, *Clinical Verification of Hearing Aid Performance*, in *Hearing Aids*, G. Popelka, et al., Editors. 2016, Springer Switzeland.
9. Nance, G. and D. Causey, *HEARING-AID EVALUATION: AN EXAMINATION OF TWO PROCEDURES.* Bulletin of Prosthetics Research - Spring, 1968.
10. Jones, I.H. and V.O. Knudsen, *What audiometry can now mean in routine practice.* Journal of the American Medical Association, 1938. **111**(7): p. 597-605.
11. Watson, N.A. and V.O. Knudsen, *Selective Amplification in Hearing Aids.* The Journal of the Acoustical Society of America, 1940. **11**(4): p. 406-419.
12. Lybarger, S., *US patent application SN543-278.* 1944.
13. Dillon, H. *The Research of Denis Byrne at NAL: Implications for Clinicians Today*. 2001 [cited 2020 25/04]; Available from: https://www.audiologyonline.com/articles/research-denis-byrne-at-nal-1200.
14. Byrne, D., et al., *NAL-NL1 procedure for fitting nonlinear hearing aids: characteristics and comparisons with other procedures.* J Am Acad Audiol, 2001. **12**(1): p. 37-51.
15. Keidser, G., et al., *The NAL-NL2 Prescription Procedure.* Audiol Res, 2011. **1**(1): p. e24.
16. Scollie, S., et al., *The Desired Sensation Level multistage input/output algorithm.* Trends Amplif, 2005. **9**(4): p. 159-97.
17. Dillon, H., *Hearing Aids*. 2012: Thieme.
18. Keidser, G., et al., *NAL-NL2 empirical adjustments.* Trends Amplif, 2012. **16**(4): p. 211-23.
19. Almufarrij, I., et al., *Direct-to-Consumer Hearing Devices: Capabilities, Costs, and Cosmetics.* Trends Hear, 2019. **23**: p. 2331216519858301.
20. Sabin, A.T., et al., *Validation of a Self-Fitting Method for Over-the-Counter Hearing Aids.* Trends Hear, 2020. **24**: p. 2331216519900589.
21. Scollie, S., et al., *Evaluation of the NAL-NL1 and DSL v4.1 prescriptions for children: Preference in real world use.* Int J Audiol, 2010. **49 Suppl 1**: p. S49-63.
22. Walravens, E., G. Keidser, and L. Hickson, *Consistency of Hearing Aid Setting Preference in Simulated Real-World Environments: Implications for Trainable Hearing Aids.* Trends Hear, 2020. **24**: p. 2331216520933392.
23. Mueller, H.G., *Fitting hearing aids to adults using prescriptive methods: an evidence-based review of effectiveness.* J Am Acad Audiol, 2005. **16**(7): p. 448-60.
24. Shamseer, L., et al., *Preferred reporting items for systematic review and meta-analysis protocols (PRISMA-P) 2015: elaboration and explanation.* Bmj, 2015. **350**: p. g7647.
25. Ventry, I.M. and B.E. Weinstein, *The hearing handicap inventory for the elderly: a new tool.* Ear Hear, 1982. **3**(3): p. 128-34.

For peer review only - http://bmjopen.bmj.com/site/about/guidelines.xhtml

26. Gatehouse, S. and W. Noble, *The Speech, Spatial and Qualities of Hearing Scale (SSQ).* International journal of audiology, 2004. **43**(2): p. 85-99.
27. Morris, S.B. and R.P. DeShon, *Combining effect size estimates in meta-analysis with repeated measures and independent-groups designs.* Psychol Methods, 2002. **7**(1): p. 105-25.

For peer review only

Appendix 1: search protocols

PubMed:

(("Hearing Loss"[Mesh] OR "hearing loss"[tw] OR "hearing losses"[tw] OR "Hearing Disorders"[Mesh] OR "hearing disorder"[tw] OR "hearing disorders"[tw] OR "hearing problem"[tw] OR "hearing problems"[tw] OR "hearing impaired"[tw] OR "hearing difficulties"[tw] OR "hearing difficulty"[tw] OR "ANSD"[tw] OR "auditory neuropathy"[tw]) AND ("Hearing Aids"[Mesh] OR "hearing aid"[tw] OR "hearing aids"[tw] OR "hearing device"[tw] OR "hearing devices"[tw] OR "acoustic aid"[tw] OR "acoustic aids"[tw] OR " hearing instrument"[tw] OR " hearing instruments"[tw] OR "over-the-counter hearing"[tw] OR "over the counter hearing "[tw] OR "direct-to-consumer hearing"[tw] OR "direct to consumer hearing"[tw] OR "self-fitting hearing"[tw] OR "self fitting hearing "[tw] OR "personal sound amplification"[tw]) AND ("real ear measurement"[tw] OR "real ear measurements"[tw] OR "real ear measure"[tw] OR "real ear measures"[tw] OR "real-ear measurement"[tw] OR "real-ear measurements"[tw] OR "real-ear measure"[tw] OR "real-ear measures"[tw] OR "REM"[tw] OR "REMs"[tw] OR "PMM"[tw] OR "PMMs"[tw] OR "probe microphone"[tw] OR "probe microphones"[tw] OR "probe mic"[tw] OR "probe mics"[tw] OR "probe microphone measurement"[tw] OR "probe microphone measurements"[tw] OR "probe microphone measure"[tw] OR "probe microphone measures"[tw] OR "probemicrophone"[tw] OR "probe-microphones"[tw] OR "probe-microphone measurement"[tw] OR "probe-microphone measurements"[tw] OR "probe-microphone measure"[tw] OR "probe-microphone measures"[tw] OR "real-ear verification"[tw] OR "audiology best practices"[tw] OR "audiology best practice"[tw] OR "best practices"[tw] OR "best practice"[tw] OR "clinician-driven"[tw] OR "physician-driven"[tw] OR "doctor-driven"[tw] OR "prescriptive methods"[tw] OR "prescription methods"[tw] OR "prescriptive method*"[tw] OR "prescription method*"[tw] OR "formula"[tw] OR "formulae"[tw]))

MEDLINE (OVID)

((exp "Hearing Loss"/ OR "hearing loss".mp OR "hearing losses".mp OR exp "Hearing Disorders"/ OR "hearing disorder".mp OR "hearing disorders".mp OR "hearing problem".mp OR "hearing problems".mp OR "hearing impaired".mp OR "hearing difficulties".mp OR "hearing difficulty".mp OR "ANSD".mp OR "auditory neuropathy".mp) AND (exp "Hearing Aids"/ OR "hearing aid".mp OR "hearing aids".mp OR "hearing device".mp OR "hearing devices".mp OR "acoustic aid".mp OR "acoustic aids".mp OR " hearing instrument".mp OR " hearing instruments".mp OR "over-the-counter hearing".mp OR "over the counter hearing ".mp OR "direct-to-consumer hearing".mp OR "direct to consumer hearing".mp OR "self-fitting hearing".mp OR "self fitting hearing ".mp OR "personal sound amplification".mp) AND ("real ear measurement".mp OR "real ear measurements".mp OR "real ear measure".mp OR "real ear measures".mp OR "real-ear measurement".mp OR "real-ear measurements".mp OR "real-ear measure".mp OR "real-ear measures".mp OR "REM".mp OR "REMs".mp OR "PMM".mp OR "PMMs".mp OR "probe microphone".mp OR "probe microphones".mp OR "probe mic".mp OR "probe mics".mp OR "probe microphone measurement".mp OR "probe microphone measurements".mp OR "probe microphone measure".mp OR "probe microphone measures".mp OR "probemicrophone".mp OR "probe-microphones".mp OR "probe-microphone measurement".mp OR "probe-microphone measurements".mp OR "probe-microphone measure".mp OR "probe-microphone measures".mp OR "real-ear verification".mp OR "audiology best practices".mp OR "audiology best practice".mp OR "best practices".mp OR "best practice".mp OR "clinician-driven".mp OR "physician-driven".mp OR "doctor-driven".mp OR "prescriptive methods".mp OR "prescription methods".mp OR "prescriptive method*".mp OR "prescription method*".mp OR "formula".mp OR "formulae".mp))

For peer review only

Embase (OVID)

((exp "Hearing Impairment"/ OR "hearing loss".mp OR "hearing losses".mp OR exp "Hearing Disorder"/ OR "hearing disorder".mp OR "hearing disorders".mp OR "hearing problem".mp OR "hearing problems".mp OR "hearing impaired".mp OR "hearing difficulties".mp OR "hearing difficulty".mp OR "ANSD".mp OR "auditory neuropathy".mp) AND (exp "Hearing Aid"/ OR "hearing aid".mp OR "hearing aids".mp OR "hearing device".mp OR "hearing devices".mp OR "acoustic aid".mp OR "acoustic aids".mp OR " hearing instrument".mp OR " hearing instruments".mp OR "over-the-counter hearing".mp OR "over the counter hearing ".mp OR "direct-to-consumer hearing".mp OR "direct to consumer hearing".mp OR "self-fitting hearing".mp OR "self fitting hearing ".mp OR "personal sound amplification".mp) AND ("real ear measurement".mp OR "real ear measurements".mp OR "real ear measure".mp OR "real ear measures".mp OR "real-ear measurement".mp OR "real-ear measurements".mp OR "real-ear measure".mp OR "real-ear measures".mp OR "REM".mp OR "REMs".mp OR "PMM".mp OR "PMMs".mp OR "probe microphone".mp OR "probe microphones".mp OR "probe mic".mp OR "probe mics".mp OR "probe microphone measurement".mp OR "probe microphone measurements".mp OR "probe microphone measure".mp OR "probe microphone measures".mp OR "probemicrophone".mp OR "probe-microphones".mp OR "probe-microphone measurement".mp OR "probe-microphone measurements".mp OR "probe-microphone measure".mp OR "probe-microphone measures".mp OR "real-ear verification".mp OR "audiology best practices".mp OR "audiology best practice".mp OR "best practices".mp OR "best practice".mp OR "clinician-driven".mp OR "physician-driven".mp OR "doctor-driven".mp OR "prescriptive methods".mp OR "prescription methods".mp OR "prescriptive method*".mp OR "prescription method*".mp OR "formula".mp OR "formulae".mp))

PsycINFO

TX(("Hearing Impairment" OR "hearing loss" OR "hearing losses" OR "Hearing Disorder" OR "hearing disorder" OR "hearing disorders" OR "hearing problem" OR "hearing problems" OR "hearing impaired" OR "hearing difficulties" OR "hearing difficulty" OR "ANSD" OR "auditory neuropathy") AND ("Hearing Aid" OR "hearing aid" OR "hearing aids" OR "hearing device" OR "hearing devices" OR "acoustic aid" OR "acoustic aids" OR " hearing instrument" OR " hearing instruments" OR "over-the-counter hearing" OR "over the counter hearing " OR "direct-to-consumer hearing" OR "direct to consumer hearing" OR "self-fitting hearing" OR "self fitting hearing " OR "personal sound amplification") AND ("real ear measurement" OR "real ear measurements" OR "real ear measure" OR "real ear measures" OR "real-ear measurement" OR "real-ear measurements" OR "real-ear measure" OR "real-ear measures" OR "REM" OR "REMs" OR "PMM" OR "PMMs" OR "probe microphone" OR "probe microphones" OR "probe mic" OR "probe mics" OR "probe microphone measurement" OR "probe microphone measurements" OR "probe microphone measure" OR "probe microphone measures" OR "probemicrophone" OR "probe-microphones" OR "probe-microphone measurement" OR "probe-microphone measurements" OR "probe-microphone measure" OR "probe-microphone measures" OR "real-ear verification" OR "audiology best practices" OR "audiology best practice" OR "best practices" OR "best practice" OR "clinician-driven" OR "physician-driven" OR "doctor-driven" OR "prescriptive methods" OR "prescription methods" OR "prescriptive method*" OR "prescription method*" OR "formula" OR "formulae"))

Web of Science (all databases)

TS=(("Hearing Impairment" OR "hearing loss" OR "hearing losses" OR "Hearing Disorder" OR "hearing disorder" OR "hearing disorders" OR "hearing problem" OR "hearing problems" OR "hearing impaired" OR "hearing difficulties" OR "hearing difficulty" OR "ANSD" OR "auditory neuropathy") AND ("Hearing Aid" OR "hearing aid" OR "hearing aids" OR "hearing device" OR "hearing devices" OR "acoustic aid" OR "acoustic aids" OR " hearing instrument" OR " hearing instruments" OR "over-the-counter hearing" OR "over the counter hearing " OR "direct-to-consumer hearing" OR "direct to consumer hearing" OR "self-fitting hearing" OR "self fitting hearing " OR "personal sound amplification") AND ("real ear measurement" OR "real ear measurements" OR "real ear measure" OR "real ear measures" OR "real-ear measurement" OR "real-ear measurements" OR "real-ear measure" OR "real-ear measures" OR "REM" OR "REMs" OR "PMM" OR "PMMs" OR "probe microphone" OR "probe microphones" OR "probe mic" OR "probe mics" OR "probe microphone measurement" OR "probe microphone measurements" OR "probe microphone measure" OR "probe microphone measures" OR "probemicrophone" OR "probe-microphones" OR "probe-microphone measurement" OR "probe-microphone measurements" OR "probe-microphone measure" OR "probe-microphone measures" OR "real-ear verification" OR "audiology best practices" OR "audiology best practice" OR "best practices" OR "best practice" OR "clinician-driven" OR "physician-driven" OR "doctor-driven" OR "prescriptive methods" OR "prescription methods" OR "prescriptive method*" OR "prescription method*" OR "formula" OR "formulae"))

Cochrane Library

(("Hearing Impairment" OR "hearing loss" OR "hearing losses" OR "Hearing Disorder" OR "hearing disorder" OR "hearing disorders" OR "hearing problem" OR "hearing problems" OR "hearing impaired" OR "hearing difficulties" OR "hearing difficulty" OR "ANSD" OR "auditory neuropathy") AND ("Hearing Aid" OR "hearing aid" OR "hearing aids" OR "hearing device" OR "hearing devices" OR "acoustic aid" OR "acoustic aids" OR " hearing instrument" OR " hearing instruments" OR "over-the-counter hearing" OR "over the counter hearing " OR "direct-to-consumer hearing" OR "direct to consumer hearing" OR "self-fitting hearing" OR "self fitting hearing " OR "personal sound amplification") AND ("real ear measurement" OR "real ear measurements" OR "real ear measure" OR "real ear measures" OR "real-ear measurement" OR "real-ear measurements" OR "real-ear measure" OR "real-ear measures" OR "REM" OR "REMs" OR "PMM" OR "PMMs" OR "probe microphone" OR "probe microphones" OR "probe mic" OR "probe mics" OR "probe microphone measurement" OR "probe microphone measurements" OR "probe microphone measure" OR "probe microphone measures" OR "probemicrophone" OR "probe-microphones" OR "probe-microphone measurement" OR "probe-microphone measurements" OR "probe-microphone measure" OR "probe-microphone measures" OR "real-ear verification" OR "audiology best practices" OR "audiology best practice" OR "best practices" OR "best practice" OR "clinician-driven" OR "physician-driven" OR "doctor-driven" OR "prescriptive methods" OR "prescription methods" OR "prescriptive method*" OR "prescription method*" OR "formula" OR "formulae")):ti,ab,kw

Emcare

((exp "Hearing Impairment"/ OR "hearing loss".mp OR "hearing losses".mp OR exp "Hearing Disorder"/ OR "hearing disorder".mp OR "hearing disorders".mp OR "hearing problem".mp OR "hearing problems".mp OR "hearing impaired".mp OR "hearing difficulties".mp OR "hearing difficulty".mp OR "ANSD".mp OR "auditory neuropathy".mp) AND (exp "Hearing Aid"/ OR "hearing aid".mp OR "hearing aids".mp OR "hearing device".mp OR "hearing devices".mp OR

For peer review only

"acoustic aid".mp OR "acoustic aids".mp OR " hearing instrument".mp OR " hearing instruments".mp OR "over-the-counter hearing".mp OR "over the counter hearing ".mp OR "direct-to-consumer hearing".mp OR "direct to consumer hearing".mp OR "self-fitting hearing".mp OR "self fitting hearing ".mp OR "personal sound amplification".mp) AND ("real ear measurement".mp OR "real ear measurements".mp OR "real ear measure".mp OR "real ear measures".mp OR "real-ear measurement".mp OR "real-ear measurements".mp OR "real-ear measure".mp OR "real-ear measures".mp OR "REM".mp OR "REMs".mp OR "PMM".mp OR "PMMs".mp OR "probe microphone".mp OR "probe microphones".mp OR "probe mic".mp OR "probe mics".mp OR "probe microphone measurement".mp OR "probe microphone measurements".mp OR "probe microphone measure".mp OR "probe microphone measures".mp OR "probemicrophone".mp OR "probe-microphones".mp OR "probe-microphone measurement".mp OR "probe-microphone measurements".mp OR "probe-microphone measure".mp OR "probe-microphone measures".mp OR "real-ear verification".mp OR "audiology best practices".mp OR "audiology best practice".mp OR "best practices".mp OR "best practice".mp OR "clinician-driven".mp OR "physician-driven".mp OR "doctor-driven".mp OR "prescriptive methods".mp OR "prescription methods".mp OR "prescriptive method*".mp OR "prescription method*".mp OR "formula".mp OR "formulae".mp))

Academic Search Premier

TI(("Hearing Impairment" OR "hearing loss" OR "hearing losses" OR "Hearing Disorder" OR "hearing disorder" OR "hearing disorders" OR "hearing problem" OR "hearing problems" OR "hearing impaired" OR "hearing difficulties" OR "hearing difficulty" OR "ANSD" OR "auditory neuropathy") AND ("Hearing Aid" OR "hearing aid" OR "hearing aids" OR "hearing device" OR "hearing devices" OR "acoustic aid" OR "acoustic aids" OR " hearing instrument" OR " hearing instruments" OR "over-the-counter hearing" OR "over the counter hearing " OR "direct-to-consumer hearing" OR "direct to consumer hearing" OR "self-fitting hearing" OR "self fitting hearing " OR "personal sound amplification") AND ("real ear measurement" OR "real ear measurements" OR "real ear measure" OR "real ear measures" OR "real-ear measurement" OR "real-ear measurements" OR "real-ear measure" OR "real-ear measures" OR "REM" OR "REMs" OR "PMM" OR "PMMs" OR "probe microphone" OR "probe microphones" OR "probe mic" OR "probe mics" OR "probe microphone measurement" OR "probe microphone measurements" OR "probe microphone measure" OR "probe microphone measures" OR "probemicrophone" OR "probe-microphones" OR "probe-microphone measurement" OR "probe-microphone measurements" OR "probe-microphone measure" OR "probe-microphone measures" OR "real-ear verification" OR "audiology best practices" OR "audiology best practice" OR "best practices" OR "best practice" OR "clinician-driven" OR "physician-driven" OR "doctor-driven" OR "prescriptive methods" OR "prescription methods" OR "prescriptive method*" OR "prescription method*" OR "formula" OR "formulae")) OR SU(("Hearing Impairment" OR "hearing loss" OR "hearing losses" OR "Hearing Disorder" OR "hearing disorder" OR "hearing disorders" OR "hearing problem" OR "hearing problems" OR "hearing impaired" OR "hearing difficulties" OR "hearing difficulty" OR "ANSD" OR "auditory neuropathy") AND ("Hearing Aid" OR "hearing aid" OR "hearing aids" OR "hearing device" OR "hearing devices" OR "acoustic aid" OR "acoustic aids" OR " hearing instrument" OR " hearing instruments" OR "over-the-counter hearing" OR "over the counter hearing " OR "direct-to-consumer hearing" OR "direct to consumer hearing" OR "self-fitting hearing" OR "self fitting hearing " OR "personal sound amplification") AND ("real ear measurement" OR "real ear measurements" OR "real ear measure" OR "real ear measures" OR "real-ear measurement" OR "real-ear measurements" OR "real-ear measure" OR "real-ear measures" OR "REM" OR "REMs" OR "PMM" OR "PMMs" OR "probe microphone" OR "probe microphones" OR "probe mic" OR "probe mics" OR "probe microphone measurement" OR "probe microphone measurements" OR "probe microphone measure" OR "probe microphone

For peer review only

measures" OR "probemicrophone" OR "probe-microphones" OR "probe-microphone measurement" OR "probe-microphone measurements" OR "probe-microphone measure" OR "probe-microphone measures" OR "real-ear verification" OR "audiology best practices" OR "audiology best practice" OR "best practices" OR "best practice" OR "clinician-driven" OR "physician-driven" OR "doctor-driven" OR "prescriptive methods" OR "prescription methods" OR "prescriptive method*" OR "prescription method*" OR "formula" OR "formulae")) OR KW(("Hearing Impairment" OR "hearing loss" OR "hearing losses" OR "Hearing Disorder" OR "hearing disorder" OR "hearing disorders" OR "hearing problem" OR "hearing problems" OR "hearing impaired" OR "hearing difficulties" OR "hearing difficulty" OR "ANSD" OR "auditory neuropathy") AND ("Hearing Aid" OR "hearing aid" OR "hearing aids" OR "hearing device" OR "hearing devices" OR "acoustic aid" OR "acoustic aids" OR " hearing instrument" OR " hearing instruments" OR "over-the-counter hearing" OR "over the counter hearing " OR "direct-to-consumer hearing" OR "direct to consumer hearing" OR "self-fitting hearing" OR "self fitting hearing " OR "personal sound amplification") AND ("real ear measurement" OR "real ear measurements" OR "real ear measure" OR "real ear measures" OR "real-ear measurement" OR "real-ear measurements" OR "real-ear measure" OR "real-ear measures" OR "REM" OR "REMs" OR "PMM" OR "PMMs" OR "probe microphone" OR "probe microphones" OR "probe mic" OR "probe mics" OR "probe microphone measurement" OR "probe microphone measurements" OR "probe microphone measure" OR "probe microphone measures" OR "probemicrophone" OR "probe-microphones" OR "probe-microphone measurement" OR "probe-microphone measurements" OR "probe-microphone measure" OR "probe-microphone measures" OR "real-ear verification" OR "audiology best practices" OR "audiology best practice" OR "best practices" OR "best practice" OR "clinician-driven" OR "physician-driven" OR "doctor-driven" OR "prescriptive methods" OR "prescription methods" OR "prescriptive method*" OR "prescription method*" OR "formula" OR "formulae")) OR AB(("Hearing Impairment" OR "hearing loss" OR "hearing losses" OR "Hearing Disorder" OR "hearing disorder" OR "hearing disorders" OR "hearing problem" OR "hearing problems" OR "hearing impaired" OR "hearing difficulties" OR "hearing difficulty" OR "ANSD" OR "auditory neuropathy") AND ("Hearing Aid" OR "hearing aid" OR "hearing aids" OR "hearing device" OR "hearing devices" OR "acoustic aid" OR "acoustic aids" OR " hearing instrument" OR " hearing instruments" OR "over-the-counter hearing" OR "over the counter hearing " OR "direct-to-consumer hearing" OR "direct to consumer hearing" OR "self-fitting hearing" OR "self fitting hearing " OR "personal sound amplification") AND ("real ear measurement" OR "real ear measurements" OR "real ear measure" OR "real ear measures" OR "real-ear measurement" OR "real-ear measurements" OR "real-ear measure" OR "real-ear measures" OR "REM" OR "REMs" OR "PMM" OR "PMMs" OR "probe microphone" OR "probe microphones" OR "probe mic" OR "probe mics" OR "probe microphone measurement" OR "probe microphone measurements" OR "probe microphone measure" OR "probe microphone measures" OR "probemicrophone" OR "probe-microphones" OR "probe-microphone measurement" OR "probe-microphone measurements" OR "probe-microphone measure" OR "probe-microphone measures" OR "real-ear verification" OR "audiology best practices" OR "audiology best practice" OR "best practices" OR "best practice" OR "clinician-driven" OR "physician-driven" OR "doctor-driven" OR "prescriptive methods" OR "prescription methods" OR "prescriptive method*" OR "prescription method*" OR "formula" OR "formulae"))

For peer review only

Appendix 2: Data items

General information	Method	Participants	Intervention	Outcome	Funding source	Declaration of interest	Additional comments
Title<br>Authors<br>Publication year	Study design<br>Conditions and the full study durations<br>Sequence generation<br>Sequence concealment<br>Blinding<br>Power analysis	Sample size<br>Country / Setting<br>Age<br>Sex<br>Inclusion and exclusion criteria	Intervention group<br>Comparator group	Primary outcome<br>Secondary outcome(s)<br>Adverse events			

For peer review only

Preferred reporting items for systematic review and meta-analysis protocols (PRISMA-P) 2015

PRISMA-P 2015 Checklist

Section/topic	Item No	Checklist item	Information reported Yes	Information reported No	Page number
**ADMINISTRATIVE INFORMATION**					
**Title**					
Identification	1a	Identify the report as a protocol of a systematic review	X		1
Update	1b	If the protocol is for an update of a previous systematic review, identify as such		X	Not an update
**Registration**	2	If registered, provide the name of the registry (e.g., PROSPERO) and registration number in the Abstract	X		2
**Authors**					
Contact	3a	Provide name, institutional affiliation, and e-mail address of all protocol authors; provide physical mailing address of corresponding author	X		1
Contributions	3b	Describe contributions of protocol authors and identify the guarantor of the review	X		11
**Amendments**	4	If the protocol represents an amendment of a previously completed or published protocol, identify as such and list changes; otherwise, state plan for documenting important protocol amendments		X	Not an amendment
**Support**					
Sources	5a	Indicate sources of financial or other support for the review	X		11
Sponsor	5b	Provide name for the review funder and/or sponsor	X		11
Role of sponsor/funder	5c	Describe roles of funder(s), sponsor(s), and/or institution(s), if any, in developing the protocol	X		11
**INTRODUCTION**					
**Rationale**	6	Describe the rationale for the review in the context of what is already known	X		4
**Objectives**	7	Provide an explicit statement of the question(s) the review will address with reference to participants, interventions, comparators, and outcomes (PICO)	X		4-5
**METHODS**					

For peer review only

**Eligibility criteria**	8	Specify the study characteristics (e.g., PICO, study design, setting, time frame) and report characteristics (e.g., years considered, language, publication status) to be used as criteria for eligibility for the review	☒	☐	5-6
**Information sources**	9	Describe all intended information sources (e.g., electronic databases, contact with study authors, trial registers, or other grey literature sources) with planned dates of coverage	☒	☐	6-7
**Search strategy**	10	Present draft of search strategy to be used for at least one electronic database, including planned limits, such that it could be repeated	☒	☐	Appendix 2
STUDY RECORDS					
Data management	11a	Describe the mechanism(s) that will be used to manage records and data throughout the review	☒	☐	7
Selection process	11b	State the process that will be used for selecting studies (e.g., two independent reviewers) through each phase of the review (i.e., screening, eligibility, and inclusion in meta-analysis)	☒	☐	7
Data collection process	11c	Describe planned method of extracting data from reports (e.g., piloting forms, done independently, in duplicate), any processes for obtaining and confirming data from investigators	☒	☐	7-8
**Data items**	12	List and define all variables for which data will be sought (e.g., PICO items, funding sources), any pre-planned data assumptions and simplifications	☒	☐	7-8
**Outcomes and prioritization**	13	List and define all outcomes for which data will be sought, including prioritization of main and additional outcomes, with rationale	☒	☐	6
**Risk of bias in individual studies**	14	Describe anticipated methods for assessing risk of bias of individual studies, including whether this will be done at the outcome or study level, or both; state how this information will be used in data synthesis	☒	☐	8
DATA					
**Synthesis**	15a	Describe criteria under which study data will be quantitatively synthesized	☒	☐	8-9
	15b	If data are appropriate for quantitative synthesis, describe planned summary measures, methods of handling data, and methods of combining data from studies, including any planned exploration of consistency (e.g., I^2, Kendall's tau)	☒	☐	9
	15c	Describe any proposed additional analyses (e.g., sensitivity or subgroup analyses, meta-regression)	☒	☐	10-11
	15d	If quantitative synthesis is not appropriate, describe the type of summary planned	☒	☐	9
**Meta-bias(es)**	16	Specify any planned assessment of meta-bias(es) (e.g., publication bias across studies, selective reporting within studies)	☒	☐	9
**Confidence in cumulative evidence**	17	Describe how the strength of the body of evidence will be assessed (e.g., GRADE)	☒	☐	10

For peer review only
